# The Performance of Large Language Models in Extracting Intestinal Symptoms From Electronic Health Records: Retrospective Observational Study

**DOI:** 10.2196/98580

**Published:** 2026-08-26

**Authors:** Xinyue Zhang, Quanyu Wang, Beibei Liu, Xinyi Sang, Sheng Wei

**Affiliations:** 1 School of Public Health and Emergency Management, Southern University of Science and Technology, 1088 Xueyuan Avenue, Shenzhen, Guangdong, 518055, China, 86 755-88011926, 86 755-88011926; 2 Department of Epidemiology and Biostatistics, Tongji Medical College, School of Public Health, Huazhong University of Science and Technology, Wuhan, Hubei, China

**Keywords:** large language models, electronic health records, intestinal infectious disease, surveillance, symptom extraction

## Abstract

**Background:**

Unstructured electronic health records (EHRs) hinder the monitoring of intestinal infections. Large language models (LLMs) enable automated symptom extraction. However, their clinical validation is limited by a lack of systematic multimodel comparisons, unclear prompting strategies, and the privacy risks of cloud-based models (eg, data leakage and cross-border data transfer).

**Objective:**

This study aimed to systematically evaluate the performance of locally deployed open-source LLMs across 4 model families in extracting intestinal symptoms from unstructured EHR chief complaints under different prompting strategies.

**Methods:**

From a citywide health care information platform in Wuhan, China, we randomly selected 1000 chief complaints from outpatient records of intestinal clinics, infectious disease departments, pediatrics, and fever clinics. Six symptoms related to intestinal infectious diseases—diarrhea/bloody/mucoid stools, vomiting, abdominal pain, fever, nausea, and rash—were manually annotated as a gold-standard dataset. Twelve locally deployed open-source LLMs across 4 families, namely, Gemma3 (1b, 4b, 12b), Qwen3 (1.7b, 8b, 14b), DeepSeek-R1 (1.5b, 7b, 14b), and Llama (Llama2-Chinese 7b, 13b; Llama3.1 8b), were evaluated on the symptom extraction task using the gold-standard dataset. Three prompting strategies (no-role, zero-shot, and few-shot) were tested. Performance metrics included accuracy, precision, recall, *F*_1_-score, specificity, balanced accuracy, and inference time. Statistical comparisons used Friedman tests for global differences, followed by Wilcoxon signed-rank and Mann-Whitney *U* tests with Bonferroni and false discovery rate corrections for pairwise comparisons.

**Results:**

Among the 4 families, Qwen3 models showed higher *F*_1_-scores and balanced accuracy, with Qwen3-1.7b achieving a macroaveraged *F*_1_-score of 0.85 under zero-shot prompting and Qwen3-8b reaching 0.89 under no-role prompting, while Gemma3 demonstrated robust performance at small to medium scales. Symptom-wise, models agreed more on frequent symptoms such as diarrhea and fever, whereas greater variability was observed for rarer symptoms like rash and nausea. The effect of prompting strategy varied across models, with no single strategy consistently outperforming the others. Although some pairwise differences reached statistical significance (*P*<.05), the absolute gains in *F*_1_-score were small.

**Conclusions:**

This study provides a systematic comparison of several open-source LLMs on a structured intestinal symptom extraction task. Among the LLM families, Qwen3 models offer a favorable balance between accuracy and efficiency, making them suitable for resource-constrained scenarios.

## Introduction

Intestinal infectious diseases (IIDs) remain a leading cause of global morbidity and mortality. Enteric infectious diseases cause substantial morbidity and mortality, disproportionately affecting children younger than 5 years of age [1]. The rapid transmission dynamics through contaminated food, water, and fomites render these infections particularly susceptible to explosive outbreaks, underscoring the imperative for robust surveillance systems capable of early detection and rapid response.

Syndromic surveillance serves as the critical sentinel for early epidemic warning. Electronic health records (EHRs) contain vast amounts of unstructured symptom data in chief complaints [2,3], representing the foundational data source for surveillance [4]. However, conventional manual extraction methods are limited by inefficiency and subjective biases [5]. Traditional natural language processing approaches for symptom extraction mainly include rule-based systems and supervised learning models. Rule-based methods rely on manually constructed regular expressions and keyword dictionaries. They are interpretable but poorly generalizable, failing to cover diverse colloquial expressions or to parse negation and conditional clauses [6]. Supervised learning models partially alleviate the generalization issue but require large expert-annotated corpora, making them costly and slow to adapt to new or rare diseases [7-9]. Despite these attempts, primary health care institutions still lack efficient and reliable automated screening tools. Consequently, the development of automated symptom extraction tools capable of delivering standardized, high-throughput screening has emerged as an imperative for enhancing prevention capabilities and enabling real-time public health intelligence [10].

Large language models (LLMs) offer a transformative solution to the limitations of traditional medical natural language processing. Through engineered prompts, LLMs such as Qwen [11] and Llama [12] can convert unstructured complaints into standardized symptom labels without escalating annotation costs, addressing critical gaps in rare disease recognition and early warning scenarios. In this study, we locally deployed open-source models, including Qwen3, DeepSeek-R1, Gemma3, Llama2-Chinese, and Llama3.1, which were adopted in Chinese clinical tasks and supported reliable local deployment. This privacy-preserving architecture is particularly critical in jurisdictions with stringent data protection regulations, where the export of personal health data to remote servers is legally prohibited [13].

This study aimed to systematically evaluate the performance of 12 locally deployed open-source LLMs across 4 model families in extracting intestinal symptoms from unstructured EHR chief complaints using no-role, zero-shot, and few-shot prompting without task-specific fine-tuning. By benchmarking diverse models and prompt strategies, we aimed to identify optimal configurations that balance timeliness with accuracy, thereby providing scientific evidence for IID symptom monitoring.

## Methods

### Study Design

We conducted a retrospective study using routinely collected EHRs from a citywide health care information platform in Wuhan, China. Outpatient records from January 1, 2023, to December 31, 2025, were included. From the eligible records, 1000 chief complaints were randomly selected to construct a gold-standard dataset through manual annotation of intestinal infection symptoms. Twelve locally deployed open-source LLMs were then evaluated under 3 prompting strategies (no-role, zero-shot, and few-shot) to assess symptom extraction performance. The study followed the STARD-AI (Standards for Reporting Diagnostic Accuracy Studies–AI) reporting guideline for diagnostic accuracy studies (Checklist 1).

### Data Sources

This study used data from the citywide health care information platform in Wuhan, which enables interoperability of health care data across all medical institutions within the city, including hospitals, community health centers, and specialized clinics. The platform captures health care records spanning outpatient visits, emergency encounters, inpatient admissions, and health screening examinations, encompassing EHRs, laboratory test results, and medication prescriptions.

A total of 167,567,887 deidentified outpatient records were obtained from the health care information platform from January 1, 2023, to December 31, 2025. All EHRs for the full calendar years were complete. Each record contained an anonymized patient identifier, clinical department, encounter timestamp, and chief complaint. Only the chief complaint field was provided to the LLMs; other fields (eg, department and timestamp) and any additional clinical notes (eg, physical examination and diagnosis) were not used for symptom extraction. An example of a chief complaint (translated from Chinese) was “fever for 2 days, diarrhea three times per day with sticky stool.”

All records were deidentified before any analysis. Regular expression matching was used to detect patterns of personally identifiable information, including patient names, phone numbers, and home addresses, and detected identifiers were replaced with masked placeholders. The unique patient identifiers were retained only as linkage keys for deduplication and were never input into the LLMs.

Given the large volume of chief complaint data in outpatient records, we initially filtered records using keyword-based filtering for terms potentially related to intestinal symptoms. Records with missing values or containing invalid entries such as “not filled,” “chief complaint unclear,” or “not specified” were excluded. Duplicate entries were then removed based on a unique identifier, onset time, and chief complaint. From the filtered dataset, we performed simple random sampling without replacement to select 1000 chief complaints for manual annotation and model evaluation. This sample size was determined by the practical constraints of manual annotation. Detailed preprocessing counts are provided in the flow diagram in Multimedia Appendix 1.

### Symptoms Related to IIDs

To identify clinical symptoms associated with IIDs, we conducted a comprehensive review of authoritative guidelines, infectious disease textbooks, and relevant studies, focusing on viral, bacterial, and parasitic enteric infections. Symptoms were initially categorized as systemic and gastrointestinal manifestations based on established case definitions (Multimedia Appendix 1). To expand and validate the symptom inventory, we conducted a systematic literature search in CNKI, Wanfang Data, PubMed, and Web of Science for papers published before June 30, 2025, using combinations of terms, including “intestinal infectious disease,” “enteric infection,” and “infectious diarrhea” paired with “syndromic surveillance.” From the retrieved literature, we compiled a database of symptoms, surveillance systems, and monitoring timelines.

### Symptom Extraction Using Locally Deployed LLMs

Twelve open-source models from 5 series were selected: Meta’s Llama-2 and Llama3.1; DeepSeek-R1; Alibaba’s Qwen3; and Google’s Gemma3, with parameter scales ranging from 1.7 billion to 14 billion. These families represent some of the most widely adopted and actively maintained open-source models. They include grouped-query attention with architectural refinements (Llama2-Chinese and Llama3.1), mixture-of-experts with multihead latent attention (DeepSeek-R1), native Chinese optimization with controllable reasoning modes (Qwen3), and sliding-window attention for multilingual efficiency (Gemma3). The selected models also cover a broad spectrum of Chinese language capability, ranging from natively Chinese-trained models to posttrained adaptations and multilingual baselines [14-16]. All are compatible with local inference frameworks, ensuring compliance with rigorous data privacy standards essential for processing EHRs in public health settings [17,18]. The selection of LLMs also reflected the practical constraints of the deployment environment. The health care platform operated on an isolated internal network with no internet access and limited graphical processing unit (GPU) resources, favoring locally deployable, quantized models.

To ensure data security, all models were deployed locally using Ollama on an Intel Xeon W-2255 processor equipped with an NVIDIA RTX A6000 GPU (48 GB VRAM) running Windows 11, version 24H2 (build 26100.4061). All models were run in standard nonthinking mode using 4-bit-quantized checkpoints (GGUF format). Inference time was measured as the end-to-end wall-clock time from sending the request to the API until the complete response was received, covering both prompt processing and output generation. All models were evaluated under identical hardware and software settings to ensure fair comparisons.

The dataset was constructed from outpatient EHRs. To evaluate symptom extraction by LLMs, we constructed a dedicated evaluation dataset. As symptoms of IIDs are mainly documented in chief complaints and cases are primarily managed in intestinal clinics, infectious disease departments, pediatrics, and fever clinics, we limited sampling to these departments to ensure coverage of target symptoms. We then randomly selected 1000 eligible records to form the model dataset without additional symptom stratification, preserving the natural distribution of symptoms in the original clinical population. Symptom labels were ascertained by 2 independent raters, both of whom held a master’s degree in public health and received training in clinical symptom annotation. Symptom-specific Cohen κ ranged from 0.87 to 1.00. The overall mean κ was 0.92 (SD 0.02). Discrepancies were resolved through consensus adjudication to establish the reference standard. Full symptom-specific κ values are provided in Multimedia Appendix 1. Annotations were completed before model inference was performed.

### Prompt Engineering

Three structured prompting strategies were evaluated: no-role prompting is defined as the baseline prompting without role definition; zero-shot prompting is defined as task instructions and output formatting without exemplars; few-shot prompting is defined as task guidance with curated input-output examples. To refine these prompts, we used a separate development set of 50 chief complaints randomly selected from the same data source, which were excluded from the final 1000-sample evaluation set. Refinement focused on ensuring output format compliance and parser robustness, without altering symptom classification criteria.

Each prompt instructed the model to return a JSON object with a predefined schema containing the symptom list. After receiving the raw text response, a rule-based parser extracted the first valid JSON object or array, validated the symptom list, and collected the symptom codes. If no parsable structure was found, the parser returned an empty list, which was treated as “no symptoms extracted.” The codes were then converted into binary indicator columns (one per symptom). Although models occasionally deviated from the required format (eg, adding extra text before or after the JSON), the parser tolerated such variations by focusing on the JSON structure. Prompt refinement procedures are provided in Multimedia Appendix 2. Batch inference was performed with fixed hyperparameters (temperature at 0.2, top-k=10, and top-p=0.5) across all models. This temperature setting was chosen to ensure a consistent comparison while allowing flexibility for nonstandard symptom expressions, consistent with prior work on clinical text mining [19,20].

### Statistical Analysis

Statistical analyses were performed to quantify performance differences across model configurations, identify key determinants of performance, and account for the hierarchical structure of the data. Model performance was evaluated at the individual symptom level relative to gold-standard manual annotations. For each of the 6 symptoms, standard binary classification metrics were computed from the confusion matrix: accuracy, precision, recall (sensitivity), specificity, balanced accuracy, and *F*_1_-score. To provide an overall summary of performance across symptoms, a macroaveraged *F*_1_-score was calculated as the unweighted mean of the 6 symptom-specific *F*_1_-scores. Computational efficiency was quantified as the average inference time per sample (in seconds per chief complaint), derived from total runtime logs for each model-prompt configuration.

Differences in performance metrics across prompting strategies (no-role, zero-shot, and few-shot) and parameter-size groups were assessed using the Friedman test on aligned symptom-level data. For significant omnibus tests (α<0.05), post hoc pairwise comparisons were performed using the Dunn test with Benjamini-Hochberg false discovery rate (FDR) correction to control for multiple testing. For inference time, the Kruskal-Wallis *H* test was used to compare parameter-size groups, with pairwise follow-up via Mann-Whitney *U* tests and FDR correction.

To disentangle the independent and interactive effects of parameter size and prompting strategy, while accounting for the hierarchical data structure (multiple symptoms nested within each model-prompt combination), linear mixed models (LMMs) were fit for each performance metric. The fixed-effects specification was defined as follows:


$$Y_{ij}=\beta_{0}+\beta_{1}\cdot Z_{ij}+\beta_{2}\cdot F_{ij}+\beta_{3}\cdot S_{j}+\beta_{4}\cdot (Z_{ij}\times S_{j})+\beta_{5}\cdot (F_{ij}\times S_{j})+\nu_{j}+\epsilon_{ij}$$

where *Y_ij_* is the performance metric for the *i*th observation within the *j*th symptom; *β*_0_ is the overall intercept; *Z_ij_* and *F_ij_* are indicator variables for prompting strategy (zero-shot and few-shot, with no-role as reference); *S_j_* is the numerical parameter size in billions; *β*_1_ to *β*_5_ are the fixed-effect coefficients for the main effects and their interaction; *υ*_*j*_~*N*(0, σ^2^_υ_) is a random intercept for each symptom, capturing symptom-level variation; *ε*_*ij*_~*N*(0, σ^2^_υ_) is the residual error.

Models were fit using restricted maximum likelihood. The significance of fixed effects was assessed using Wald tests. All statistical analyses and visualizations were performed using Python software (version 3.12.7). Two-tailed tests were used with a significance level of α=.05.

### Ethical Considerations

This retrospective study used deidentified EHRs from a citywide health care information platform in Wuhan, China, and did not involve human interventions or the collection of sensitive personal information. The requirement for informed consent was waived because all data were anonymized prior to analysis. All analyses were conducted on a secure institutional server; only aggregate statistics were reported, and no patient identifiers appeared in any figures, tables, or appendices. The study was conducted in accordance with the Declaration of Helsinki and the Measures for the Ethical Review of Life Sciences and Medical Research Involving Humans (2023, China), under which ethical review is waived for research using anonymized data that does not involve human participants or sensitive personal information.

## Results

### Symptoms Identified

We selected studies focused on IID symptom monitoring and extracted information on study titles, symptoms, surveillance systems, and study timelines. In total, we included 172 related papers in the final review (Multimedia Appendix 3). Drawing from surveillance programs, treatment guidelines, expert consensus, and research findings, we selected 6 representative core symptoms for IIDs. These included 3 gastrointestinal symptoms (diarrhea/bloody/mucoid stools, vomiting, and abdominal pain) and 3 systemic symptoms (fever, nausea, and rash), the details of which are provided in Multimedia Appendix 3.

### Overall Performance and Efficiency

Table 1 summarizes global performance across all model-prompt configurations, presenting descriptive statistics, including macro *F*_1_-score, balanced accuracy, and inference time. Additional metrics are provided in Multimedia Appendix 4. The results show variation in performance across different models, parameter sizes, and prompting strategies. Among all configurations, Qwen3-8b with no-role prompting achieved a macro *F*_1_-score of 0.89 and balanced accuracy of 0.95±0.06, with an inference time of 3.64 seconds per record, whereas Qwen3-1.7b under zero-shot prompting achieved a macro *F*_1_-score of 0.85 with a faster inference time of 1.61 seconds. By comparison, Llama3.1-8b achieved macro *F*_1_-scores of 0.35 to 0.40 across prompting strategies, with substantially longer inference times (4.29-11.74 s). Inference time also varied substantially, with larger models generally requiring more processing time per record. This aligns with expectations for the computational resource requirements of larger LLM configurations.

**Table 1.** Macro *F*_1_-score, balanced accuracy, and inference time for each model and prompting strategy.

Model and prompt	Macro *F*_1_-score	Balanced accuracy, mean (SD)	Time (seconds/record)
Qwen3-1.7b			
No-role	0.63	0.85 (0.13)	1.40
Zero-shot	0.85	0.91 (0.09)	1.61
Few-shot	0.83	0.91 (0.08)	1.52
Qwen3-8b			
No-role	0.89	0.95 (0.06)	3.64
Zero-shot	0.78	0.85 (0.11)	6.06
Few-shot	0.82	0.88 (0.09)	8.04
Qwen3-14b			
No-role	0.85	0.92 (0.09)	12.80
Zero-shot	0.82	0.89 (0.09)	10.13
Few-shot	0.87	0.91 (0.08)	11.23
DeepSeek-R1-1.5b			
No-role	0.11	0.53 (0.05)	2.84
Zero-shot	0.32	0.76 (0.13)	2.91
Few-shot	0.36	0.73 (0.12)	2.75
DeepSeek-R1-7b			
No-role	0.40	0.68 (0.18)	3.34
Zero-shot	0.74	0.87 (0.10)	3.17
Few-shot	0.70	0.90 (0.10)	2.55
DeepSeek-R1-14b			
No-role	0.63	0.79 (0.19)	8.32
Zero-shot	0.70	0.94 (0.08)	7.05
Few-shot	0.73	0.94 (0.08)	19.83
Gemma3-1b			
No-role	0.51	0.81 (0.10)	0.20
Zero-shot	0.19	0.61 (0.08)	0.69
Few-shot	0.27	0.68 (0.11)	0.63
Gemma3-4b			
No-role	0.51	0.81 (0.10)	0.66
Zero-shot	0.55	0.92 (0.06)	0.61
Few-shot	0.58	0.86 (0.08)	0.55
Gemma3-12b			
No-role	0.77	0.96 (0.04)	0.32
Zero-shot	0.63	0.95 (0.05)	1.07
Few-shot	0.77	0.94 (0.06)	1.13
Llama2-Chinese-7b			
No-role	0.00	0.50 (0.00)	13.91
Zero-shot	0.03	0.50 (0.01)	2.68
Few-shot	0.11	0.57 (0.08)	6.04
Llama2-Chinese-13b			
No-role	0.00	0.50 (0.00)	7.14
Zero-shot	0.24	0.65 (0.09)	4.88
Few-shot	0.27	0.78 (0.12)	1.74
Llama3-8b			
No-role	0.35	0.88 (0.07)	4.29
Zero-shot	0.40	0.91 (0.06)	7.20
Few-shot	0.37	0.90 (0.06)	11.74

### Performance and Inference Time by Model

**Figure 1.**
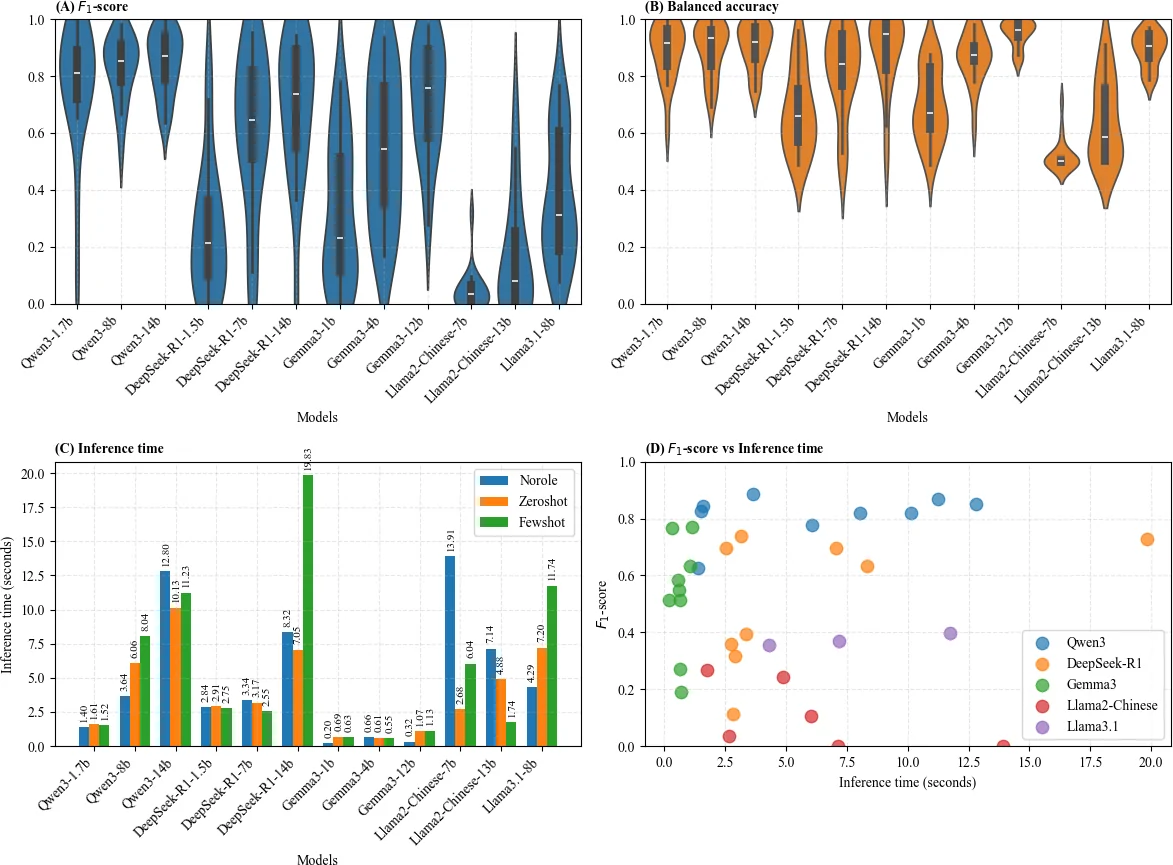
Performance and efficiency of large language models for symptom extraction as a function of parameter size. (A) *F*_1_-score distribution across all symptoms and prompts for each model. (B) Balanced accuracy distribution across all symptoms and prompts for each model. (C) Inference time (seconds per 1000 chief complaints) for each model under 3 prompting strategies: no-role (baseline instruction), zero-shot (task description without examples), and few-shot (with input-output exemplars). (D) *F*_1_-score vs inference time; each point represents one model-prompt combination. Points are colored by model family and series (Qwen3, DeepSeek-R1, Gemma3, Llama2-Chinese, and Llama3.1). Model families are arranged from left to right in order of increasing parameter size within each family.

The relationship between model scale, performance, and computational cost across all configurations is summarized in Figure 1. Violin plots of symptom-level macro *F*_1_-scores (Figure 1A) and balanced accuracy (Figure 1B) reveal different distribution patterns across model families. Qwen3 models, especially the 8b and 14b variants, exhibited high and narrow distributions for both metrics, indicating stable performance. Llama2-Chinese models exhibited lower distributions. Llama3.1-8b showed modestly higher and slightly narrower distributions than Llama2-Chinese, but remained below Qwen3 models. Inference time increased nonlinearly with parameter size and prompting strategy (Figure 1C). Among the largest models, Qwen3-14b required approximately 12.80 seconds per record under no-role prompting, while DeepSeek-R1-14b required 19.83 seconds under few-shot prompting, the longest latency observed. Gemma3 models maintained consistently short inference times regardless of scale or prompting condition. The efficiency-accuracy trade-off is further illustrated in the scatter plot (Figure 1D). Gemma3 models cluster toward the left side, reflecting faster inference times, whereas Qwen3 models occupy higher positions, indicating higher *F*_1_-scores. DeepSeek-R1 models were more dispersed, with smaller variants falling in the mid-range and the 14b few-shot setting achieving higher accuracy at the cost of longer inference. Llama3.1-8b appears in the lower-right region, reflecting its moderate *F*_1_-scores and long inference times, while Llama2-Chinese models occupy the lower-left region with low *F*_1_-scores and relatively short inference times. The performance ranking, integrating normalized *F*_1_-scores with processing times, is detailed in Multimedia Appendix 4.

### Performance Across Prompting Strategies Within Models

**Figure 2.**
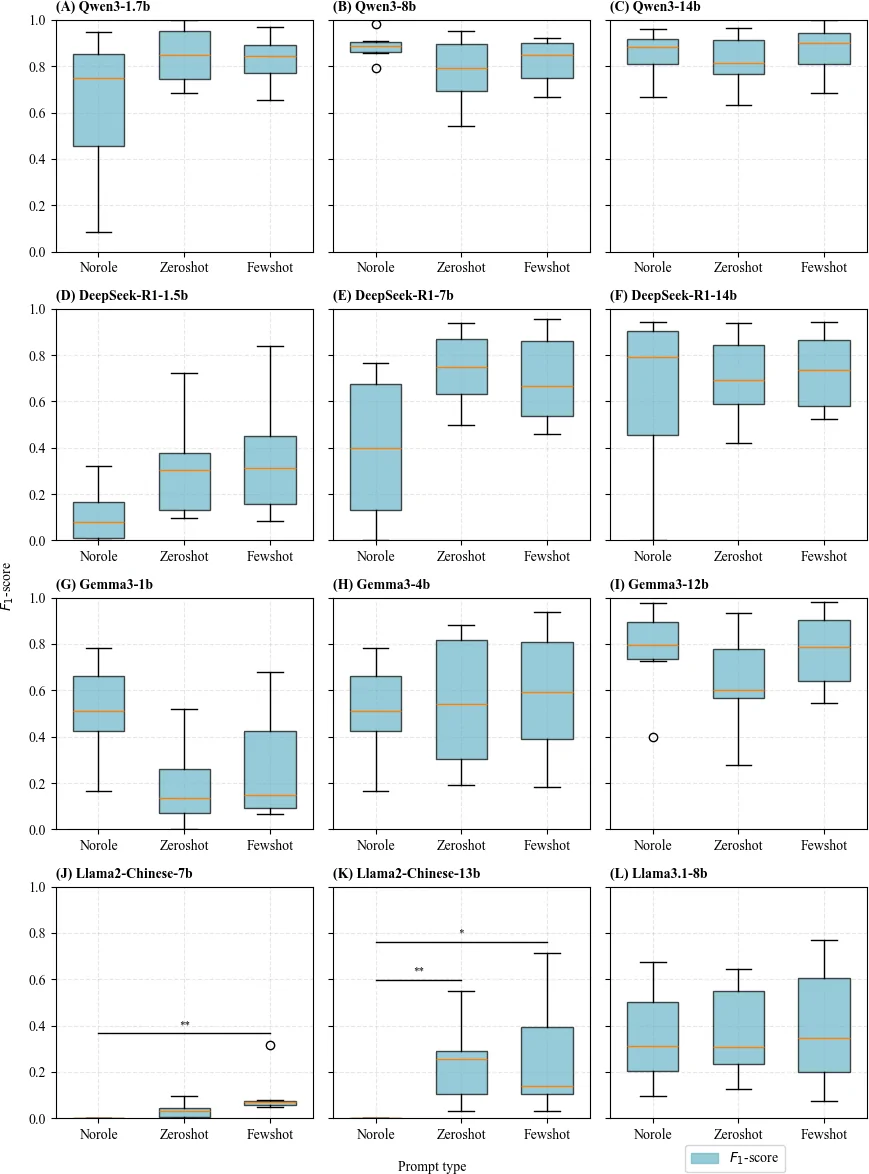
Impact of prompting strategy on *F*_1_-score across individual models. Box plots show the *F*_1_-score for 3 prompting strategies (no-role, zero-shot, and few-shot) for each model. Significant differences between prompt pairs are indicated by lines above the boxes: *P<.05, **P<.01, ***P<.001. Nonsignificant comparisons are not marked.

*F*_1_-scores across the 3 prompting strategies for each model are shown in Figure 2. The effect of prompting strategy was model-dependent, and no single approach dominated across all models. Qwen3-8b achieved its highest *F*_1_-score of 0.89 with no-role prompting, while its few-shot and zero-shot scores were 0.82 and 0.78, respectively. DeepSeek-R1-7b performed best with zero-shot prompting (*F*_1_-score=0.74), and DeepSeek-R1-14b maintained relatively stable performance across all 3 prompt types. Llama3.1-8b achieved its highest *F*_1_-score of 0.40 under zero-shot prompting, while no-role and few-shot exhibited lower or similar scores. For models with low baseline performance (eg, Llama2-Chinese-7b), none of the prompts improved the *F*_1_-score beyond 0.11. These findings indicated that the optimal prompting strategy varied by model family and that relying solely on prompt engineering cannot reliably guarantee improved performance, while model architecture and parameter scale appeared to exert an influence on symptom extraction accuracy.

### Comparison of Key Configurations

Overall, our results revealed a significant effect of prompting strategy on most performance metrics (*F*_1_-score: *χ*^2^_2_=163.9; *P*<.001; balanced accuracy: *χ*^2^_2_=151.8; *P*<.001). When averaged across symptoms (macro *F*_1_-scores), the differences between prompting strategies were small and inconsistent (Multimedia Appendix 4). Pairwise comparisons (Table 2) showed that the larger-size parameter groups were slower than smaller parameter groups (FDR-adjusted *P*<.05). Within the DeepSeek-R1 family, the 14b model significantly outperformed the 1.5b model in accuracy and specificity under both no-role and zero-shot prompts. In the Gemma3 family, the 12b model surpassed the 1b model across multiple metrics, particularly under few-shot prompting. For Llama2-Chinese, the 13b model showed better recall and balanced accuracy than the 7b model under few-shot and zero-shot prompts. Detailed pairwise results are provided in Table 2.

**Table 2.** Significant pairwise comparisons of prompting strategies and parameter sizes with respect to performance^a^.

Family: comparison models	Metric	Direction
DeepSeek-R1: 1.5b vs 14b		
No-role	Accuracy	14b>1.5b
No-role	Specificity	14b>1.5b
Zero-shot	Accuracy	14b>1.5b
Zero-shot	Specificity	14b>1.5b
Gemma3: 1b vs 12b		
Few-shot	*F*_1_-score	12b>1b
Few-shot	Balanced accuracy	12b>1b
Few-shot	Accuracy	12b>1b
Few-shot	Specificity	12b>1b
No-role	Balanced accuracy	12b>1b
No-role	Recall	12b>1b
Zero-shot	*F*_1_-score	12b>1b
Zero-shot	Balanced accuracy	12b>1b
Zero-shot	Accuracy	12b>1b
Llama2-Chinese: 7b vs 13b		
Few-shot	Recall	13b>7b
Zero-shot	Balanced accuracy	13b>7b
Zero-shot	Recall	13b>7b

a Only comparisons with FDR-adjusted *P*<.05 are shown. The Mann-Whitney *U* test was applied to each contrast. Within-family comparisons used the smallest and largest parameter sizes available within that family (DeepSeek-R1: 1.5b vs 14b; Gemma3: 1b vs 12b; Llama2-Chinese: 7b vs 13b).

### Symptom-Specific Performance

**Figure 3.**
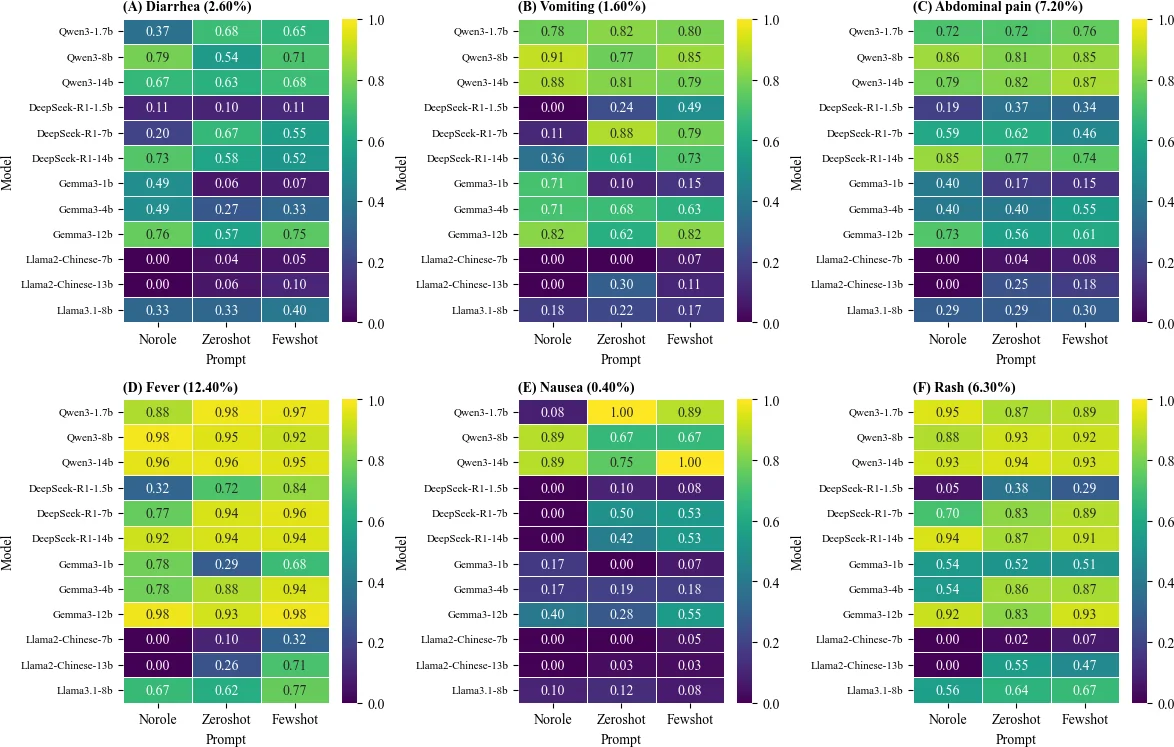
Heatmaps of *F*_1_-score for individual symptoms. Each panel corresponds to 1 of the 6 symptoms: (A) diarrhea, (B) vomiting, (C) abdominal pain, (D) fever, (E) nausea, and (F) rash. Color intensity reflects the *F*_1_-score, ranging from 0 to 1. Models are ordered by family and increasing parameter size. The heatmaps reveal substantial variation in symptom-level performance across models and prompting strategies.

Figure 3 shows *F*_1_-scores for each of the 6 symptoms across models and prompting strategies. The most challenging symptom was nausea, with *F*_1_-scores below 0.5 for all models except Qwen3-8b and Qwen3-1.7b under few-shot prompting. Diarrhea was identified with high accuracy (*F*_1_-score>0.8) by most models. The few-shot prompting strategy improved recognition of less frequent symptoms (eg, rash), whereas the no-role strategy often failed to detect them (*F*_1_-score<0.1 for the Llama2-Chinese family). These patterns suggest that few-shot examples help the model generalize to underrepresented symptom classes. Confusion matrices for all models across 6 target symptoms are detailed in Multimedia Appendix 5.

### Effect of Parameter Size on Performance Metrics

**Figure 4.**
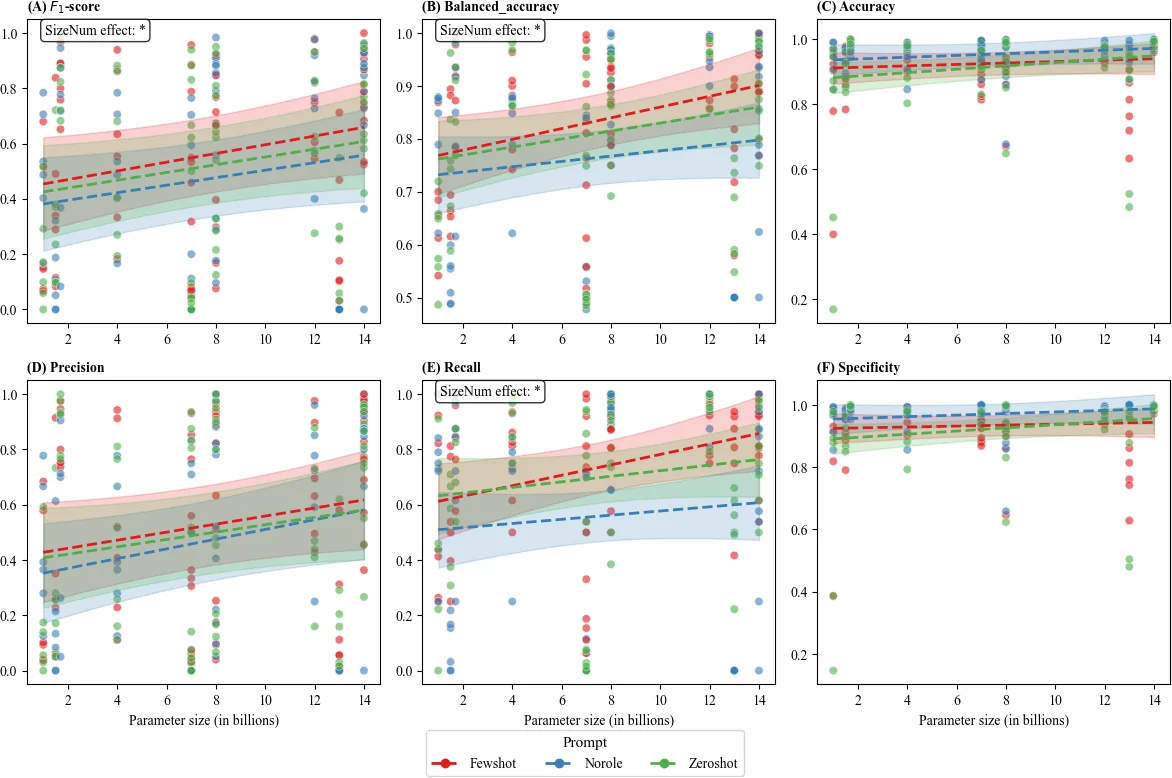
Effect of parameter size on performance metrics. Scatter plots showing the relationship between parameter size (in billions) and performance metrics across 3 prompting strategies (no-role, zero-shot, and few-shot). Solid lines represent fixed-effects predictions from linear mixed-effects models (LMMs) with random intercepts for symptoms, including the main effect of parameter size, prompting strategy, and their interaction. Shaded areas indicate 95% CIs for the predictions: (A) *F*_1_-score, (B) balanced accuracy, (C) accuracy, (D) precision, (E) recall, and (F) specificity. Asterisks denote the significance of the parameter size main effect (LMM: *P<.05, **P<.01, ***P<.001).

Linear mixed-model analysis showed the effect of parameter size on performance metrics, as illustrated in Figure 4. Under few-shot prompting, each 1 billion increase in parameter size was associated with an increase of 0.0102 in balanced accuracy (95% CI 0.0023‐0.0180). Similarly, recall improved by 0.0189 per 1 billion (95% CI 0.0034‐0.0344). Trends toward improvement were observed for precision, though this did not reach conventional significance (β=0.0146, 95% CI −0.0015 to 0.0307; β=0.0075, 95% CI −0.0007 to 0.0157). Other metrics (accuracy, *F*_1_-score, and specificity) did not show a statistically significant association with parameter size (all *P*>.05). Full model results, including coefficients and 95% CIs, are provided in Multimedia Appendix 4.

## Discussion

### Principal Findings

This study systematically evaluated 12 open-source LLMs across 4 model families (Qwen3, DeepSeek-R1, Gemma3, and Llama) for extracting intestinal symptoms from EHRs. Qwen3-8b with no-role prompting achieved the highest macroaveraged *F*_1_-score of 0.89 with a moderate inference time of 3.64 seconds per record. Although no universal *F*_1_-score threshold exists for clinical deployment, prior studies have considered scores between 0.80 and 0.84 acceptable for similar tasks [21]. Our observed *F*_1_-score of 0.89, combined with a recall of 0.91 and precision of 0.88, suggests potential for population-level surveillance. Prospective validation in real-time settings and use-case–specific calibration are needed before full deployment.

### Prompting Strategy Effects Varied Across Tasks and Models

The findings revealed that the effect of prompting strategy varied across models, with no single strategy consistently outperforming the others. Although some pairwise comparisons reached statistical significance, the absolute differences in metrics were small, and the direction of improvement was not consistent across models.

The effect of prompting strategies was most pronounced for the small models in our evaluation, such as Qwen3-1.7b and DeepSeek-R1-1.5b, where zero-shot and few-shot prompting produced more noticeable gains than for larger models. This observation aligns with a broader trend in the literature: prompting techniques tend to yield larger relative improvements in smaller language models, where the baseline zero-shot performance is lower, and the parameter space is more limited [22,23]. For larger models, the absolute differences among prompting strategies were smaller, suggesting that their stronger pretrained representations may already capture much of the task-relevant information without additional in-context guidance [24]. From a practical perspective, this finding has important implications for resource-constrained deployments, and our results suggest that prompt design can partially compensate for their smaller parameter capacity. At the same time, the modest effect of prompting strategies across most models indicates that deploying LLMs in such settings may not require extensive prompt optimization. Concise, straightforward instructions may be adequate.

These results contrast with previous studies demonstrating substantial gains from few-shot prompting in clinical settings [25,26], but align with recent work showing that the effectiveness of prompting strategies is highly model-dependent. For instance, Sivarajkumar et al [27] found that while heuristic and chain-of-thought prompts excelled in certain tasks, no single prompting strategy universally outperformed the others across the 5 clinical tasks examined. Similarly, a systematic review concluded that applying performance-improvement strategies, including few-shot prompting, may, in some cases, even degrade performance [28]. The model-dependent nature of prompting effects may suggest that the benefits of sophisticated prompt engineering may be modest for binary classification tasks such as symptom extraction [29]. Alternatively, it may indicate that the few-shot exemplars used were insufficient to capture the nuanced patterns across the target symptoms [30].

### Performance Disparities by Symptom Prevalence

The analysis revealed that extraction performance varied considerably by symptom type. LLMs pretrained on standard clinical corpora may therefore have weaker representations for symptoms with variable expressions. The lower extraction accuracy for rare symptoms reflects inherent bias in pretraining corpora, which plays a foundational role. LLMs learn statistical regularities from vast text collections that reflect real-world prevalence patterns. Common symptoms such as fever and abdominal pain are overrepresented in medical literature and clinical notes, giving models internal representations for frequent entities [31]. Rarer symptoms achieve substantially lower extraction accuracy. As a previous study demonstrated, class imbalance and missingness of signs and symptoms systematically degrade model performance [32]. Xi et al [26] observed that symptom extraction remains the most challenging entity type in rare disease named entity recognition because symptom labels are context-dependent and often overlap with objective findings, exacerbating the impact of data scarcity.

Model architecture and pretraining data composition matter more than parameter count for rare symptom recognition. McMurry et al [33] further demonstrated that while LLMs significantly outperformed *International Classification of Diseases*, Tenth Revision (*ICD-10*)–based methods for respiratory symptom identification, performance variability across symptoms persisted, with rarer symptoms remaining more difficult to extract. Our finding that Qwen3-1.7b outperformed several larger models suggests that model architecture is important for rare symptom recognition. For surveillance systems targeting rare or emerging symptoms, additional strategies such as targeted data augmentation or retrieval-augmented generation may be needed to complement prompt-based approaches. Our prompt-based workflow is model-agnostic and requires no code modification when switching between LLMs, offering a readily deployable solution that can leverage future improved models.

### Parameter-Size Improvements in Balanced Accuracy

LLMs revealed that models with larger parameter sizes primarily enhance sensitivity, the ability to correctly identify true symptoms. Previous studies have demonstrated that increasing model size improves generalization and reasoning capabilities, which may translate into better identification of symptoms in clinical text [33]. The significant effect on recall is particularly important in clinical contexts where missing a symptom may have more serious consequences than false alarms [34].

The time-to-completion analysis revealed a substantial trade-off between performance and inference efficiency, with larger models incurring markedly longer inference times. The 14b models required an average of 11.6 seconds per chief complaint, compared to 0.5 seconds for 1b models. The findings align with a previous study, which demonstrated that medium-sized Llama models (7b-8b) achieve competitive performance while running up to 28 times faster than 70b counterparts [31]. This efficiency gap underscores the practical constraints of deploying deep learning models in resource-limited clinical settings [35] and highlights the potential of medium-sized models to balance accuracy with throughput.

However, inference time did not always increase with model size. For short-context classification tasks, inference latency is not solely determined by parameter count. Hybrid attention mechanisms, quantization, and runtime optimizations can enable larger models to run faster than smaller, less efficient ones. These observations suggest that model selection for deployment in resource-constrained settings should consider not only parameter size but also architectural efficiency and quantization compatibility.

### Model Architecture Matters More Than Parameter Size Alone

Our findings reveal that model architecture and training paradigms exert a stronger influence on symptom recognition performance than parameter size alone. While increasing parameter count generally improves performance, the gains exhibit diminishing returns, with the difference between 8b and 14b models failing to reach statistical significance in our analysis. This pattern aligns with recent observations in medical AI evaluation, where architectural innovations can yield substantial performance advantages that are not solely attributable to model scale [36].

The poor performance of Llama2-Chinese further underscores this point. Llama2-Chinese was only posttrained on general Chinese dialogue data, not on medical corpora. Effective adaptation to the Chinese clinical domain reportedly requires continued pretraining. This pattern also extended to Llama3.1-8b, which achieved macro *F*_1_-scores that were modestly better than those of Llama2-Chinese but still below the performance of Qwen3 models. These findings suggest that Llama-series models may be less suitable for the Chinese intestinal symptom extraction task.

We further observed that model architecture can influence performance as profoundly as parameter count. In our symptom extraction task, medium-sized Qwen3 models (1.7b and 8b) outperformed larger models from Llama2-Chinese. Similar patterns have emerged in other clinical information extraction studies, which reported that when extracting social determinants of health from EHRs, open-source models such as OpenChat-3.5 (approximately 7b parameters) consistently outperformed the baseline, while comparably sized Llama-2 models exhibited inferior performance [34]. Collectively, these observations indicate that parameter size alone does not determine accuracy; architectural design, domain relevance of pretraining data, and instruction-tuning strategies are also critical [37]. For unstructured tasks such as clinical notes, well-engineered medium-sized models can achieve deployable performance in resource-constrained settings, whereas simply increasing model size may yield diminishing returns [38-40].

### Clinical Implications

Our local deployment strategy ensures that patient data remain within the institutions, countering the privacy risks associated with commercial health data brokerage [41]. By avoiding external data transmission, this approach upholds patient confidentiality while maintaining competitive model performance, offering a sustainable pathway for AI integration that aligns with emerging regulatory frameworks prioritizing data transparency and patient autonomy. The findings suggest that for straightforward symptom extraction tasks, moderate-sized models (3-8b parameters) may offer the optimal balance between accuracy and efficiency.

Deployment decisions should balance extraction performance and inference time according to the specific clinical workflow, whether processing is done in real time or in batches, and the acceptable latency for the intended use case. Our inference measurements were performed with a batch size of 1 to reflect single-record latency. In practice, when processing high volumes of data, batch processing can substantially reduce the average time per record by amortizing model initialization and GPU kernel launch overhead. For retrospective symptom extraction in an outpatient clinic, the Qwen3-8b model may be appropriate given its higher *F*_1_-score. For real-time applications such as emergency department triage or outbreak alert systems, lower latency may be necessary. Alternatively, the faster Qwen3-1.7b model offers a favorable trade-off between speed and accuracy.

The limited and model-dependent nature of prompting effects also has practical implications. For symptom classification tasks, clinicians and researchers may not need to invest substantial effort in prompt optimization, as simple, clear instructions appear sufficient to achieve near-optimal performance in some configurations. This observation aligns with recent findings in clinical text classification [42].

### Limitations

Several limitations warrant consideration. First, the absence of external validation across different clinical settings or languages constrains the conclusions about model generalizability. Our results may not extend to non-Chinese EHRs, to other health care institutions with different documentation practices, or to symptom categories other than intestinal symptoms (eg, respiratory or neurological symptoms) [34]. Second, we only used the chief complaint field, which is typically short. Symptom extraction from longer clinical notes, such as the history of present illness or physical examination, may yield different performance and should be investigated in future work. In addition, precision and *F*_1_-score capture false positives but do not explicitly quantify hallucination rates (ie, the proportion of extracted symptoms not mentioned in the input text); future work should incorporate dedicated hallucination metrics to assess clinical trustworthiness. Furthermore, the lack of domain-specific fine-tuning means our results represent out-of-the-box performance [43]. We did not perform fine-tuning because our study focused on evaluating the capability of open-source general LLMs for symptom extraction under realistic conditions where rapid deployment without task-specific training is required. This workflow is model-agnostic and can be reapplied to future LLMs without code modification or additional annotation. Finally, the time analysis was conducted on a specific hardware configuration; inference times on other hardware (especially newer GPUs) and across different deployment environments may differ substantially and should be interpreted with caution.

Future work should extend the evaluation to more diverse symptom types and clinical settings, and leverage increasingly capable general-purpose LLMs with richer medical pretraining, which can be integrated into our workflow. Our pipeline is positioned to integrate such newer open-source general-purpose LLMs without additional fine-tuning, offering a sustainable path for keeping pace with rapid LLM advances. Retrieval-augmented generation approaches may further reduce hallucinations and improve grounding.

### Conclusions

In this systematic evaluation of open-source LLMs for intestinal symptom extraction, we found that model architecture and parameter size significantly influenced accuracy, with Qwen models offering a favorable trade-off between performance and inference efficiency. Few-shot prompting provided modest gains for rare symptoms but did not consistently outperform simpler instructions across all models or metrics. These findings suggest that for binary symptom classification, moderate-sized models (3-8b) with clear baseline prompts may be sufficient for many resource-constrained clinical deployments.

## Supplementary material

10.2196/98580 Multimedia Appendix 1 Symptoms of intestinal infectious diseases from clinical guidelines.

10.2196/98580 Multimedia Appendix 2 Refinement process for extracting target symptoms in a large language model.

10.2196/98580 Multimedia Appendix 3 Identification of target symptoms for intestinal infectious diseases.

10.2196/98580 Multimedia Appendix 4 Statistics for all models.

10.2196/98580 Multimedia Appendix 5 Confusion matrices for all models across the 6 target symptoms.

10.2196/98580 Checklist 1 STARD-AI checklist.

## Abbreviations

EHR: electronic health record
FDR: false discovery rate
GPU: graphical processing unit
*ICD-10*: *International Classification of Diseases*, Tenth Revision
IID: intestinal infectious disease
LLM: large language model
LMM: linear mixed model
STARD-AI: Standards for Reporting Diagnostic Accuracy Studies–AI
