# Supplementary material for: The Performance of Large Language Models in Extracting Intestinal Symptoms From Electronic Health Records: Retrospective Observational Study
Source: J Med Internet Res. 2026 Aug 26;28:e98580. doi: 10.2196/98580 (PMC13509444; doi:10.2196/98580)
Supplement: Supplementary file 1 [file jmir-v28-e98580-s006.pdf]

Section and Topic	No.	STARD-AI Item	Reported on page
**Title or abstract**			
	1	Identification as a study reporting AI-centred diagnostic accuracy and reporting at least one measure of accuracy within title or abstract	2
**Abstract**			
	2	Structured summary of study design, methods, results and conclusions (for specific guidance, please see STARD for Abstracts)	2
**Introduction**			
	3	Scientific and clinical background, including the intended use of the index test, whether it is novel or an established index test, and its integration into an existing or new workflow, if applicable	3, 4
	4	Study objectives and hypotheses	4
**Methods**			
Study design	5	Whether data collection was planned before the index test and reference standard were performed (prospective study) or after (retrospective study)	4
Ethics	6	Formal approval from an ethics committee. If not required, justify why	9
Participants	7	Eligibility criteria: listing separate inclusion and exclusion criteria in the order that they are applied at both participant level and data level	5
	8	On what basis potentially eligible participants were identified (such as symptoms, results from previous tests, inclusion in registry)	5
	9	Where and when potentially eligible participants were identified (setting, location, and dates)	4, 5
	10	Whether participants formed a consecutive, random, or convenience series	5
Dataset	11	Source of the data and whether it has been routinely collected, specifically collected for the purpose of the study or acquired from an open-source repository	4, 5
	12	Who undertook the annotations for the dataset (including experience levels and background) and how (within the same clinical context or in a post-hoc fashion), if applicable	7
	13	Devices (manufacturer, model) that were used to capture data; software (with version number) used to engineer the index test, highlighting the intended use	6
	14	Data acquisition protocols (e.g. contrast protocol or reconstruction method for medical images) and details of data pre- processing in sufficient detail to allow replication	4, 5
Test methods	15a	Index test, in sufficient detail to allow replication	5, 6
	15b	How the index test was developed, including any training, validation, testing and external evaluation, detailing sample sizes, when applicable	6;<br>Multimedia Appendix 1
	15c	Definition of and rationale for test positivity cut-offs or result categories of the index test, distinguishing pre-specified from exploratory	7
	15d	The specified end user of the index test and the level of expertise required of users	7
	16a	Reference standard, in sufficient detail to allow replication	7

	16b	Rationale for choosing the reference standard (if alternatives exist)	5, 6
	16c	Definition of and rationale for test positivity cut-offs or result categories of the reference standard, distinguishing pre-specified from exploratory	5, 6
	17a	Whether clinical information and reference standard results were available to the performers or readers of the index test	7
	17b	Whether clinical information and index test results were available to the assessors of the reference standard	7
Analysis	18	Methods for estimating or comparing measures of diagnostic accuracy	8
	19	How indeterminate index test or reference standard results were handled	Not applicable.
	20	How missing data on the index test and reference standard were handled	No missing data for the chief complaint field remained after preprocessing.
	21	Any analyses of variability in diagnostic accuracy, distinguishing pre-specified from exploratory	8, 9
	22	Intended sample size and how it was determined	5
	23	Details of any performance error analysis, and algorithmic bias and fairness assessments if undertaken	Error analysis was performed by stratifying performance by symptom prevalence (see Discussion). Bias/fairness was not formally assessed; potential generalisability limitations are acknowledged (see Limitations).
**Results**			
Participants and dataset	24	Flow of participants, using a diagram	Multimedia Appendix 1
	25	Baseline demographic, clinical and technical characteristics of training, validation and test set, if applicable	Figure 3
	26a	Distribution of severity of disease in those with the target condition	Not applicable.
	26b	Distribution of alternative diagnoses in those without the target condition	Not applicable.
	27	Time interval and any clinical interventions between index test and reference standard	Index test and reference standard were both applied to the same static chief complaint text; no time interval or clinical interventions occurred.
	28	Whether the datasets represent the distribution of the target condition that one would expect from the intended use population	4, 5
	29	For external evaluation on an independent dataset, an assessment of how this differs from the training, validation and test sets	Not applicable.
Test results	30	Cross tabulation of the index test results (or their distribution) by the results of the reference standard	Table 1;<br>Figure3;<br>Multimedia Appendix 4
	31	Estimates of diagnostic accuracy and their precision (such as 95% confidence intervals)	Table 1<br>Multimedia Appendix 4

	32	Any adverse events from performing the index test or the reference standard	Not applicable.
**Discussion**			
	33	Study limitations, including sources of potential bias, statistical uncertainty, and generalisability	23, 24
	34	Implications for practice, including the intended use and clinical role of the index test	19-24
	35	Ethical considerations and adherence to ethical standards associated with the use of the index test and issues of fairness	9, 10, 24, 25
**Other information**			
	36	Registration number and name of registry	Not applicable
	37	Where the full study protocol can be accessed	Not applicable
	38	Sources of funding and other support; role of funders	25
	39	Commercial interests, if applicable	25
	40a	Availability of datasets and code; detailing any restrictions on their reuse and repurposing	25
	40b	Whether outputs are stored, auditable and available for evaluation, if necessary	Available upon reasonable request
