# Supplementary material for: The Performance of Large Language Models in Extracting Intestinal Symptoms From Electronic Health Records: Retrospective Observational Study
Source: J Med Internet Res. 2026 Aug 26;28:e98580. doi: 10.2196/98580 (PMC13509444; doi:10.2196/98580)
Supplement: Supplementary file 2 [file jmir-v28-e98580-s001.docx]

**Table 1. Target symptoms of intestinal infectious diseases from clinical guidelines.**

| **Disease** | **Intestinal Symptoms** | **Systemic Symptoms** | **Source** |
| --- | --- | --- | --- |
| **Viral diarrhea** | Vomiting, abdominal pain, diarrhea, and abnormal stool patterns (watery or loose stools) | nausea | <https://www.chinacdc.cn/jkyj/crb2/bl/grxfxb/jswj_fxb/202409/P020240907592486658798.pdf> |
| **Infectious diarrhea** | Diarrhea, abnormal stool patterns, vomiting, abdominal pain | Fever, nausea, loss of appetite and general discomfort | <https://www.who.int/publications/i/item/9789240098985>；  <https://www.ncbi.nlm.nih.gov/pubmed/29889925>;  <https://www.nhc.gov.cn/wjw/s9491/200704/38817/files/1739781344388_99393.pdf> |
| **Rotavirus gastroenteritis** | Vomiting, diarrhea | Fever, nausea | <https://www.chinacdc.cn/jkyj/crb2/bl/grxfxb/jswj_fxb/202409/P020240907592485015438.pdf> |
| **Enterovirus 71(EV71) infection** | Vomiting | Fever, nausea, rash, cough, runny nose, loss of appetite, headache | <https://www.chinacdc.cn/jkyj/crb2/bl/grxfxb/jswj_fxb/202409/P020240907592483436518.pdf> |
| **Cholera** | Diarrhea, abdominal discomfort, abnormal stool patterns, bloody stool, vomiting | Fever | <https://www.nhc.gov.cn/cms-search/downFiles/ae3d98d3d36a4f15b2e4b1dcb2ee879e.pdf> |
| **Dysentery** | Abdominal pain, diarrhea, tenesmus, mucus and bloody stool | - | <https://www.nhc.gov.cn/wjw/s9491/200802/39040/files/1739781367464_91241.pdf>;  <https://www.who.int/publications/i/item/9241592330> |
| **Poliomyelitis** | Diarrhea or constipation | Fever, nausea, discomfort in the throat, excessive sweating, muscle aches, etc | <https://www.nhc.gov.cn/wjw/s9491/201606/55fca4a0e4b0458bba1659fda26c22e6.shtml> |
| **Typhoid fever and paratyphoid fever** | Digestive tract symptoms | Fever, relatively slow pulse, roseola, hepatosplenomegaly, etc | <https://www.nhc.gov.cn/wjw/s9491/200802/38811.shtml> |
| **Norovirus infection** | Diarrhea and vomiting, followed by abdominal pain | Headache, fever, nausea, chills and muscle aches | <https://www.nhc.gov.cn/wjw/zsdw/201511/807b70bed9fd4e80983d92bba0e8a075.shtml> |
| **Herpangina** | Vomiting, diarrhea, sometimes accompanied by abdominal pain | Fever, sore throat, cough, runny nose, sometimes accompanied by headache | <https://rs.yiigle.com/cmaid/1117392> |
| **Hand foot and mouth disease** | - | Fever, rash, may be accompanied by symptoms such as cough, runny nose and loss of appetite | <https://www.gov.cn/zhengce/zhengceku/2018-12/31/content_5435156.htm> |

**Notes:**

"Abnormal stool patterns" includes watery stool, loose stool, mucoid stool, etc.

"-" indicates no relevant symptoms documented.

**Figure 1. Flowchart of chief complaint data cleaning.**

The process involved two main steps: (1) keyword-based filtering to discard invalid entries (e.g., "not filled", "unclear", "not specified"); (2) keyword-based filtering to retain records mentioning intestinal symptoms and deduplication by hospital visit ID, onset time, and chief complaint to eliminate redundant records.

**Table 2. Inter-annotator agreement for each symptom.**

| **Symptom** | **Kappa** | **95% CI** |
| --- | --- | --- |
| **Diarrhea** | 0.89 | 0.78, 0.98 |
| **Vomiting** | 0.87 | 0.73, 0.97 |
| **Abdominal pain** | 0.86 | 0.79, 0.92 |
| **Fever** | 0.96 | 0.94, 0.99 |
| **Nausea** | 1.00 | 1.00, 1.00 |
| **Rash** | 0.94 | 0.89, 0.98 |
