# Supplementary material for: The Performance of Large Language Models in Extracting Intestinal Symptoms From Electronic Health Records: Retrospective Observational Study
Source: J Med Internet Res. 2026 Aug 26;28:e98580. doi: 10.2196/98580 (PMC13509444; doi:10.2196/98580)
Supplement: Supplementary file 3 [file jmir-v28-e98580-s002.docx]

**Table 1. Versions, formats, and deployment details of large language models.**

| Model Family | Model Name | Version/Tag | Format | Deployment Method |
| --- | --- | --- | --- | --- |
| Qwen3 | Qwen3-1.7b | qwen3:1.7b | GGUF | Ollama run qwen3:1.7b |
|  | Qwen3-8b | qwen3:8b | GGUF | Ollama run qwen3:8b |
|  | Qwen3-14b | qwen3:14b | GGUF | Ollama run qwen3:14b |
| Deepseek-r1 | Deepseek-r1-1.5b | deepseek-r1:1.5b | GGUF | Ollama run deepseek-r1:1.5b |
|  | Deepseek-r1-7b | deepseek-r1:7b | GGUF | Ollama run deepseek-r1:7b |
|  | Deepseek-r1-14b | deepseek-r1:14b | GGUF | Ollama run deepseek-r1:14b |
| Gemma3 | Gemma3-1b | gemma3:1b | GGUF | Ollama run gemma3:1b |
|  | Gemma3-4b | gemma3:4b | GGUF | Ollama run gemma3:4b |
|  | Gemma3-12b | gemma3:12b | GGUF | Ollama run gemma3:12b |
| Llama2-Chinese | Llama2-Chinese-7b | llama2-chinese:7b | GGUF | Ollama run llama2-chinese:7b |
|  | Llama2-Chinese-13b | llama2-chinese:13b | GGUF | Ollama run llama2-chinese:13b |
| Llama3.1 | Llama3.1-8b | llama3.1:8b | GGUF | Ollama run llama3.1:8b |

All models were downloaded from the Ollama registry (https://ollama.com/library) using the ollama pull <model:tag> command. Deployment was performed locally on the same hardware configuration described in the Methods section.

**Table 2. Refinement process for extracting target symptoms in large language model.**

| Character | Content |
| --- | --- |
| User | In order to identify symptoms based on chief complaints, we target a total of six symptoms: 1) diarrhea, 2) emesis, 3) abdominal pain, 4) fever, 5) nausea, and 6) rash. Symptoms should be strictly identified based on the chief complaint. A target symptom is only confirmed if it's explicitly stated or specific standard synonyms, like 'diarrhoea' for 'loose stools', 'vomiting and diarrhoea' for 'vomiting and diarrhoea', 'fever' for 'fever', and 'rash' for 'rash', are used: [text]. |
| System | Please answer in a fixed format. |
| User | Identifying Symptoms Based on Patients' Chief Complaints, with a Total of Six Target Symptoms: 1) diarrhea, 2) emesis, 3) abdominal pain, 4) fever, 5) nausea, and 6) rash.  Please pay special attention to the following matters:  Strictly identify symptoms based on the chief complaints. A target symptom is only confirmed if it's explicitly stated or specific standard synonyms are used, such as 'loose stools' for 'diarrhoea', 'vomiting and diarrhoea' for both 'vomiting' and 'diarrhoea', 'fever' for 'pyrexia', and 'rash onset' for 'rash'.  Never infer symptoms from vague expressions, assumptions, associations, or disease names. Here are some examples to clarify the boundaries:  1.Direct descriptions of stool consistency changes like 'loose stools', 'watery stools', 'mucous stools', 'bloody stools', 'unformed stools', 'hematochezia', 'blood in stool', or 'watery stools' should be identified as 'diarrhoea'. Don't infer other symptoms. 'Diarrhoea' must only be identified as 'diarrhoea', not as 'abdominal pain'.  2.Expressions such as 'abnormal stools', 'difficulty passing stool', or 'stool changes' that don't directly describe stool consistency shouldn't be identified as 'changes in stool consistency'. Similarly, terms like 'hard stools' describing dry stools shouldn't be identified as 'diarrhoea'.  3.Terms like 'feeling nauseous' or 'wanting to vomit' that only describe sensations shouldn't be identified as 'vomiting'. However, if terms such as 'vomiting' (indicating actual vomiting behavior) are used, they should be identified as 'vomiting', not 'nausea'.  4.Common expressions for abdominal pain, such as 'upper abdominal discomfort', 'epigastric pain', 'periumbilical pain', 'dull abdominal pain', or 'abdominal discomfort', should all be identified as 'abdominal pain'. But expressions like 'menstrual abdominal pain', 'abdominal pain due to trauma', or 'postoperative abdominal pain' that explicitly indicate the cause of abdominal pain (unrelated to intestinal infections) or negations like 'no significant abdominal pain' shouldn't be identified as 'abdominal pain'.  5.Negations such as 'fever subsided', 'no fever', or 'no pyrexia' shouldn't be identified as 'fever'. However, body temperatures above 37.3°C or low-grade fevers should be identified as 'fever'.  6.Symptoms such as nausea, vomiting, or abdominal pain caused by non-intestinal infections (e.g., pregnancy, early pregnancy, alcohol use, sexual activity, amenorrhea, intravenous infusion, or menstruation) shouldn't be identified as such.  7.Common expressions for rash onset, such as 'rash', 'skin rash', 'eruption', 'red rash', 'vesicular rash', or 'papular rash', should all be identified as 'rash'. But negations like 'no rash' or 'rash resolved' shouldn't be identified as 'rash'. Rashes from skin conditions like psoriasis, atopic dermatitis, acne, herpes zoster, or urticaria, or from sexually transmitted diseases like condyloma acuminatum, or allergic reactions to topical medications, IV infusions, or surgeries, shouldn't be identified as 'rash'.  Chief complaint text to analyze:  {clinical_note_text} |
| System | Please answer in a fixed format. |
| User | Identifying Symptoms Based on Patients' Chief Complaints, with a Total of Six Target Symptoms: 1) diarrhea, 2) emesis, 3) abdominal pain, 4) fever, 5) nausea, and 6) rash.  Please pay special attention to the following matters:  Strictly identify symptoms based on the chief complaints. A target symptom is only confirmed if it's explicitly stated or specific standard synonyms are used, such as 'loose stools' for 'diarrhoea', 'vomiting and diarrhoea' for both 'vomiting' and 'diarrhoea', 'fever' for 'pyrexia', and 'rash onset' for 'rash'.  Never infer symptoms from vague expressions, assumptions, associations, or disease names. Here are some examples to clarify the boundaries:  1.Direct descriptions of stool consistency changes like 'loose stools', 'watery stools', 'mucous stools', 'bloody stools', 'unformed stools', 'hematochezia', 'blood in stool', or 'watery stools' should be identified as 'diarrhoea'. Don't infer other symptoms. 'Diarrhoea' must only be identified as 'diarrhoea', not as 'abdominal pain'.  2.Expressions such as 'abnormal stools', 'difficulty passing stool', or 'stool changes' that don't directly describe stool consistency shouldn't be identified as 'changes in stool consistency'. Similarly, terms like 'hard stools' describing dry stools shouldn't be identified as 'diarrhoea'.  3.Terms like 'feeling nauseous' or 'wanting to vomit' that only describe sensations shouldn't be identified as 'vomiting'. However, if terms such as 'vomiting' (indicating actual vomiting behavior) are used, they should be identified as 'vomiting', not 'nausea'.  4.Common expressions for abdominal pain, such as 'upper abdominal discomfort', 'epigastric pain', 'periumbilical pain', 'dull abdominal pain', or 'abdominal discomfort', should all be identified as 'abdominal pain'. But expressions like 'menstrual abdominal pain', 'abdominal pain due to trauma', or 'postoperative abdominal pain' that explicitly indicate the cause of abdominal pain (unrelated to intestinal infections) or negations like 'no significant abdominal pain' shouldn't be identified as 'abdominal pain'.  5.Negations such as 'fever subsided', 'no fever', or 'no pyrexia' shouldn't be identified as 'fever'. However, body temperatures above 37.3°C or low-grade fevers should be identified as 'fever'.  6.Symptoms such as nausea, vomiting, or abdominal pain caused by non-intestinal infections (e.g., pregnancy, early pregnancy, alcohol use, sexual activity, amenorrhea, intravenous infusion, or menstruation) shouldn't be identified as such.  7.Common expressions for rash onset, such as 'rash', 'skin rash', 'eruption', 'red rash', 'vesicular rash', or 'papular rash', should all be identified as 'rash'. But negations like 'no rash' or 'rash resolved' shouldn't be identified as 'rash'. Rashes from skin conditions like psoriasis, atopic dermatitis, acne, herpes zoster, or urticaria, or from sexually transmitted diseases like condyloma acuminatum, or allergic reactions to topical medications, IV infusions, or surgeries, shouldn't be identified as 'rash'.  If the same target symptom is mentioned more than once in the chief complaint, output it only once.  If the chief complaint contains only phrases like 'same history as before' or 'examination', which don't mention any target symptoms or their standard synonyms, don't identify any target symptoms, even if they might be inferred.  If the chief complaint includes content that appears related to target symptoms but isn't a standard expression or synonym (e.g., 'feeling a bit off, but no specific discomfort described'), don't identify any target symptoms.  Chief complaint text to analyze:  {clinical_note_text} |
| System | Please respond in a fixed format. |
| User | Identifying Symptoms Based on Patients' Chief Complaints, with a Total of Six Target Symptoms: 1) diarrhea, 2) emesis, 3) abdominal pain, 4) fever, 5) nausea, and 6) rash.  Please pay special attention to the following matters:  Strictly identify symptoms based on the chief complaints. A target symptom is only confirmed if it's explicitly stated or specific standard synonyms are used, such as 'loose stools' for 'diarrhoea', 'vomiting and diarrhoea' for both 'vomiting' and 'diarrhoea', 'fever' for 'pyrexia', and 'rash onset' for 'rash'.  Never infer symptoms from vague expressions, assumptions, associations, or disease names. Here are some examples to clarify the boundaries:  1.Direct descriptions of stool consistency changes like 'loose stools', 'watery stools', 'mucous stools', 'bloody stools', 'unformed stools', 'hematochezia', 'blood in stool', or 'watery stools' should be identified as 'diarrhoea'. Don't infer other symptoms. 'Diarrhoea' must only be identified as 'diarrhoea', not as 'abdominal pain'.  2.Expressions such as 'abnormal stools', 'difficulty passing stool', or 'stool changes' that don't directly describe stool consistency shouldn't be identified as 'changes in stool consistency'. Similarly, terms like 'hard stools' describing dry stools shouldn't be identified as 'diarrhoea'.  3.Terms like 'feeling nauseous' or 'wanting to vomit' that only describe sensations shouldn't be identified as 'vomiting'. However, if terms such as 'vomiting' (indicating actual vomiting behavior) are used, they should be identified as 'vomiting', not 'nausea'.  4.Common expressions for abdominal pain, such as 'upper abdominal discomfort', 'epigastric pain', 'periumbilical pain', 'dull abdominal pain', or 'abdominal discomfort', should all be identified as 'abdominal pain'. But expressions like 'menstrual abdominal pain', 'abdominal pain due to trauma', or 'postoperative abdominal pain' that explicitly indicate the cause of abdominal pain (unrelated to intestinal infections) or negations like 'no significant abdominal pain' shouldn't be identified as 'abdominal pain'.  5.Negations such as 'fever subsided', 'no fever', or 'no pyrexia' shouldn't be identified as 'fever'. However, body temperatures above 37.3°C or low-grade fevers should be identified as 'fever'.  6.Symptoms such as nausea, vomiting, or abdominal pain caused by non-intestinal infections (e.g., pregnancy, early pregnancy, alcohol use, sexual activity, amenorrhea, intravenous infusion, or menstruation) shouldn't be identified as such.  7.Common expressions for rash onset, such as 'rash', 'skin rash', 'eruption', 'red rash', 'vesicular rash', or 'papular rash', should all be identified as 'rash'. But negations like 'no rash' or 'rash resolved' shouldn't be identified as 'rash'. Rashes from skin conditions like psoriasis, atopic dermatitis, acne, herpes zoster, or urticaria, or from sexually transmitted diseases like condyloma acuminatum, or allergic reactions to topical medications, IV infusions, or surgeries, shouldn't be identified as 'rash'.  If the same target symptom is mentioned more than once in the chief complaint, output it only once.  If the chief complaint contains only phrases like 'same history as before' or 'examination', which don't mention any target symptoms or their standard synonyms, don't identify any target symptoms, even if they might be inferred.  If the chief complaint includes content that appears related to target symptoms but isn't a standard expression or synonym (e.g., 'feeling a bit off, but no specific discomfort described'), don't identify any target symptoms.  Here is an example:  Input: I have been vomiting and suffering from diarrhoea, with loose stools, headache, rash on hands and feet accompanied by coughing, as well as nasal congestion  The symptoms correspond to: "vomiting and diarrhoea" for "2 Vomiting" and "3 Diarrhoea" with codes 2 and 3; "loose stools" for "1 Diarrhoea" with code 1; and "rash" for "6 Rash" with code 6.  Chief complaint text to analyze:  {clinical_note_text} |
| System | Please respond in a fixed format. For example, if the identified target symptom is fever, the response should be "Target symptom number is: 4." If no target symptoms are identified, the answer should be "No target symptoms identified." |
| User  (No role Prompt) | Task Description:  - From the provided clinical chief complaint, extract information for the following six target symptoms: diarrhea, vomiting, abdominal pain, fever, nausea, rash.  - For each symptom, determine its status, provide the supporting text snippet, and indicate its starting and ending character positions in the chief complaint.  Output Format:  Return a JSON object. Each key must be one of the six symptom names (in English). The value for each key must be another JSON object containing the following fields:  - "status": One of three strings – "Affirmed", "Negated", or "Absent".  - "text": If status is "Affirmed" or "Negated", this field is required and must contain the exact text snippet from the chief complaint supporting your judgment. If status is "Absent", this field should be an empty string "".  - "start_char": The starting character index of the supporting text snippet (integer, 0‑based).  - "end_char": The ending character index of the supporting text snippet (integer, pointing to the position after the last character, convenient for slicing).  Status Judgment Rules:  1. Focus on recent experience: The judgment should be based on the patient's symptoms experienced in the days before the visit. As long as the chief complaint mentions that a symptom occurred recently, even if it has resolved or improved by the time of the visit, it should be judged as "Affirmed".  2. Status definitions:  - "Affirmed": The symptom is clearly described as present. This includes phrases like "abdominal pain for 2 days", "accompanied by fever", "self-reported nausea", "vomited once", "diarrhea", etc. Even if the symptom is "improving" or "alleviating", it is still considered affirmed. For uncertain expressions like "suspected" or "possible", as long as they indicate the symptom's existence, they are treated as affirmed.  - "Negated": The symptom is explicitly stated as absent. This includes negative words such as "no", "denies", "not seen", "not reported". For example, "no abdominal pain", "denies fever".  - "Absent": The chief complaint contains neither affirmation nor negation of the symptom; it is not mentioned at all.  3. Conflict handling: If both affirmation and negation appear for the same symptom (e.g., "had diarrhea yesterday, but no diarrhea today"), the recent occurrence takes precedence and should be judged as "Affirmed".  4. Symptom-specific identification tips:  - Diarrhea: Look for terms like "diarrhea", "loose stools", "watery stool", "increased stool frequency". Changes in stool consistency are important clues.  - Vomiting / nausea: Look for "vomiting", "nausea", "dry heaving", "threw up". The abbreviation "n/v" is occasionally used in Chinese records to indicate nausea and vomiting.  - Abdominal pain: Look for "abdominal pain", "stomach ache", "abdominal discomfort", "colic", "dull pain", "periumbilical pain", "lower abdominal pain".  - Fever: Look for "fever", "high temperature", "chills", "rigors" (often accompanying fever).  - Rash: Look for "rash", "skin eruption", "erythema", "papules", "urticaria", "redness".  Chief complaint text to analyze:  {clinical_note_text} |
| System | Please respond in a fixed format. For example, if the identified target symptom is fever, the response should be "Target symptom number is: 4." If no target symptoms are identified, the answer should be "No target symptoms identified." |
| User  (Zero-shot Prompt) | Role Setting:  You are an experienced public health expert and clinical language processing specialist. Your task is to accurately extract six target symptoms related to intestinal infectious diseases from the chief complaint text of electronic medical records.  Task Description:  - From the provided clinical chief complaint, extract information for the following six target symptoms: diarrhea, vomiting, abdominal pain, fever, nausea, rash.  - For each symptom, determine its status, provide the supporting text snippet, and indicate its starting and ending character positions in the chief complaint.  Output Format:  Return a JSON object. Each key must be one of the six symptom names (in English). The value for each key must be another JSON object containing the following fields:  - "status": One of three strings – "Affirmed", "Negated", or "Absent".  - "text": If status is "Affirmed" or "Negated", this field is required and must contain the exact text snippet from the chief complaint supporting your judgment. If status is "Absent", this field should be an empty string "".  - "start_char": The starting character index of the supporting text snippet (integer, 0‑based).  - "end_char": The ending character index of the supporting text snippet (integer, pointing to the position after the last character, convenient for slicing).  Status Judgment Rules:  1. Focus on recent experience: The judgment should be based on the patient's symptoms experienced in the days before the visit. As long as the chief complaint mentions that a symptom occurred recently, even if it has resolved or improved by the time of the visit, it should be judged as "Affirmed".  2. Status definitions:  - "Affirmed": The symptom is clearly described as present. This includes phrases like "abdominal pain for 2 days", "accompanied by fever", "self-reported nausea", "vomited once", "diarrhea", etc. Even if the symptom is "improving" or "alleviating", it is still considered affirmed. For uncertain expressions like "suspected" or "possible", as long as they indicate the symptom's existence, they are treated as affirmed.  - "Negated": The symptom is explicitly stated as absent. This includes negative words such as "no", "denies", "not seen", "not reported". For example, "no abdominal pain", "denies fever".  - "Absent": The chief complaint contains neither affirmation nor negation of the symptom; it is not mentioned at all.  3. Conflict handling: If both affirmation and negation appear for the same symptom (e.g., "had diarrhea yesterday, but no diarrhea today"), the recent occurrence takes precedence and should be judged as "Affirmed".  4. Symptom-specific identification tips:  - Diarrhea: Look for terms like "diarrhea", "loose stools", "watery stool", "increased stool frequency". Changes in stool consistency are important clues.  - Vomiting / nausea: Look for "vomiting", "nausea", "dry heaving", "threw up". The abbreviation "n/v" is occasionally used in Chinese records to indicate nausea and vomiting.  - Abdominal pain: Look for "abdominal pain", "stomach ache", "abdominal discomfort", "colic", "dull pain", "periumbilical pain", "lower abdominal pain".  - Fever: Look for "fever", "high temperature", "chills", "rigors" (often accompanying fever).  - Rash: Look for "rash", "skin eruption", "erythema", "papules", "urticaria", "redness".  Chief complaint text to analyze:  {clinical_note_text} |
| System | Please respond in a fixed format. For example, if the identified target symptom is fever, the response should be "Target symptom number is: 4." If no target symptoms are identified, the answer should be "No target symptoms identified." |
| User  (Few-shot Prompt) | Role Setting:  You are an experienced public health expert and clinical language processing specialist. Your task is to accurately extract six target symptoms related to intestinal infectious diseases from the chief complaint text of electronic medical records.  Task Description:  - From the provided clinical chief complaint, extract information for the following six target symptoms: diarrhea, vomiting, abdominal pain, fever, nausea, rash.  - For each symptom, determine its status, provide the supporting text snippet, and indicate its starting and ending character positions in the chief complaint.  Output Format:  Return a JSON object. Each key must be one of the six symptom names (in English). The value for each key must be another JSON object containing the following fields:  - "status": One of three strings – "Affirmed", "Negated", or "Absent".  - "text": If status is "Affirmed" or "Negated", this field is required and must contain the exact text snippet from the chief complaint supporting your judgment. If status is "Absent", this field should be an empty string "".  - "start_char": The starting character index of the supporting text snippet (integer, 0‑based).  - "end_char": The ending character index of the supporting text snippet (integer, pointing to the position after the last character, convenient for slicing).  Status Judgment Rules:  1. Focus on recent experience: The judgment should be based on the patient's symptoms experienced in the days before the visit. As long as the chief complaint mentions that a symptom occurred recently, even if it has resolved or improved by the time of the visit, it should be judged as "Affirmed".  2. Status definitions:  - "Affirmed": The symptom is clearly described as present. This includes phrases like "abdominal pain for 2 days", "accompanied by fever", "self-reported nausea", "vomited once", "diarrhea", etc. Even if the symptom is "improving" or "alleviating", it is still considered affirmed. For uncertain expressions like "suspected" or "possible", as long as they indicate the symptom's existence, they are treated as affirmed.  - "Negated": The symptom is explicitly stated as absent. This includes negative words such as "no", "denies", "not seen", "not reported". For example, "no abdominal pain", "denies fever".  - "Absent": The chief complaint contains neither affirmation nor negation of the symptom; it is not mentioned at all.  3. Conflict handling: If both affirmation and negation appear for the same symptom (e.g., "had diarrhea yesterday, but no diarrhea today"), the recent occurrence takes precedence and should be judged as "Affirmed".  4. Symptom-specific identification tips:  - Diarrhea: Look for terms like "diarrhea", "loose stools", "watery stool", "increased stool frequency". Changes in stool consistency are important clues.  - Vomiting / nausea: Look for "vomiting", "nausea", "dry heaving", "threw up". The abbreviation "n/v" is occasionally used in Chinese records to indicate nausea and vomiting.  - Abdominal pain: Look for "abdominal pain", "stomach ache", "abdominal discomfort", "colic", "dull pain", "periumbilical pain", "lower abdominal pain".  - Fever: Look for "fever", "high temperature", "chills", "rigors" (often accompanying fever).  - Rash: Look for "rash", "skin eruption", "erythema", "papules", "urticaria", "redness".  Examples (for reference, showing output format and reasoning logic):  Example 1  Chief complaint: 腹泻水样便3天，无恶心呕吐。  Output:  {  "diarrhea": {"status": "Affirmed", "text": "腹泻水样便3天", "start_char": 0, "end_char": 7},  "vomiting": {"status": "Negated", "text": "无恶心呕吐", "start_char": 8, "end_char": 13},  "abdominal pain": {"status": "Absent", "text": "", "start_char": -1, "end_char": -1},  "fever": {"status": "Absent", "text": "", "start_char": -1, "end_char": -1},  "nausea": {"status": "Negated", "text": "无恶心呕吐", "start_char": 8, "end_char": 13},  "rash": {"status": "Absent", "text": "", "start_char": -1, "end_char": -1}  }  Example 2  Chief complaint: 发热伴腹痛2天，无皮疹。  Output:  {  "diarrhea": {"status": "Absent", "text": "", "start_char": -1, "end_char": -1},  "vomiting": {"status": "Absent", "text": "", "start_char": -1, "end_char": -1},  "abdominal pain": {"status": "Affirmed", "text": "腹痛2天", "start_char": 3, "end_char": 7},  "fever": {"status": "Affirmed", "text": "发热伴腹痛2天", "start_char": 0, "end_char": 7},  "nausea": {"status": "Absent", "text": "", "start_char": -1, "end_char": -1},  "rash": {"status": "Negated", "text": "无皮疹", "start_char": 8, "end_char": 11}  }  Example 3  Chief complaint: 恶心呕吐半天，疑似腹泻。  Output:  {  "diarrhea": {"status": "Affirmed", "text": "疑似腹泻", "start_char": 5, "end_char": 9},  "vomiting": {"status": "Affirmed", "text": "恶心呕吐半天", "start_char": 0, "end_char": 6},  "abdominal pain": {"status": "Absent", "text": "", "start_char": -1, "end_char": -1},  "fever": {"status": "Absent", "text": "", "start_char": -1, "end_char": -1},  "nausea": {"status": "Affirmed", "text": "恶心呕吐半天", "start_char": 0, "end_char": 6},  "rash": {"status": "Absent", "text": "", "start_char": -1, "end_char": -1}  }  Example 4  Chief complaint: 腹痛阵作2天，今日已无腹痛，仍感恶心。  Output:  {  "diarrhea": {"status": "Absent", "text": "", "start_char": -1, "end_char": -1},  "vomiting": {"status": "Absent", "text": "", "start_char": -1, "end_char": -1},  "abdominal pain": {"status": "Affirmed", "text": "腹痛阵作2天", "start_char": 0, "end_char": 6},  "fever": {"status": "Absent", "text": "", "start_char": -1, "end_char": -1},  "nausea": {"status": "Affirmed", "text": "仍感恶心", "start_char": 13, "end_char": 17},  "rash": {"status": "Absent", "text": "", "start_char": -1, "end_char": -1}  }  Chief complaint text to analyze:  {clinical_note_text} |
| System | Please respond in a fixed format. For example, if the identified target symptom is fever, the response should be "Target symptom number is: 4." If no target symptoms are identified, the answer should be "No target symptoms identified." |
