# Supplementary material for: The Performance of Large Language Models in Extracting Intestinal Symptoms From Electronic Health Records: Retrospective Observational Study
Source: J Med Internet Res. 2026 Aug 26;28:e98580. doi: 10.2196/98580 (PMC13509444; doi:10.2196/98580)
Supplement: Supplementary file 4 [file jmir-v28-e98580-s003.docx]

**Figure 1.** Flowchart of researches review for intestinal infectious diseases symptom surveillance.

Records identified through database searching (PubMed, Web of Science, CNKI, Wanfang Data Knowledge Service Platform) through 30 June 2025**.** A total of 172 studies were included in the final review after duplicate removal, title/abstract screening, and full-text assessment.

**Figure 2. Identification of key symptoms for intestinal infectious diseases syndromic surveillance.**
