# Supplementary material for: The Performance of Large Language Models in Extracting Intestinal Symptoms From Electronic Health Records: Retrospective Observational Study
Source: J Med Internet Res. 2026 Aug 26;28:e98580. doi: 10.2196/98580 (PMC13509444; doi:10.2196/98580)
Supplement: Supplementary file 5 [file jmir-v28-e98580-s004.docx]

**Table 1. Full descriptive statistics (mean, standard deviation, median) for all metrics by model and prompt.**

|  |  | **Accuracy** | | | **Precision** | | | **Specificity** | | | **Recall** | | | **F1-score** | | |
| --- | --- | --- | --- | --- | --- | --- | --- | --- | --- | --- | --- | --- | --- | --- | --- | --- |
| **Model** | **Prompt** | **mean** | **std** | **median** | **mean** | **std** | **median** | **mean** | **std** | **median** | **mean** | **std** | **median** | **mean** | **std** | **median** |
| Qwen3-1.7b | norole | 0.97 | 0.02 | 0.97 | 0.59 | 0.36 | 0.71 | 0.98 | 0.02 | 0.98 | 0.71 | 0.26 | 0.78 | 0.63 | 0.33 | 0.75 |
|  | zeroshot | 0.99 | 0.01 | 0.99 | 0.90 | 0.10 | 0.93 | 1.00 | 0.01 | 1.00 | 0.82 | 0.18 | 0.85 | 0.85 | 0.13 | 0.85 |
|  | fewshot | 0.99 | 0.01 | 0.99 | 0.83 | 0.11 | 0.78 | 0.99 | 0.01 | 1.00 | 0.84 | 0.15 | 0.86 | 0.83 | 0.11 | 0.84 |
| Qwen3-8b | norole | 0.99 | 0.01 | 0.99 | 0.88 | 0.09 | 0.85 | 0.99 | 0.01 | 1.00 | 0.91 | 0.13 | 0.95 | 0.89 | 0.06 | 0.89 |
|  | zeroshot | 0.99 | 0.01 | 0.99 | 0.92 | 0.07 | 0.93 | 1.00 | 0.00 | 1.00 | 0.70 | 0.22 | 0.74 | 0.78 | 0.16 | 0.79 |
|  | fewshot | 0.99 | 0.01 | 0.99 | 0.92 | 0.06 | 0.93 | 1.00 | 0.00 | 1.00 | 0.76 | 0.18 | 0.84 | 0.82 | 0.11 | 0.85 |
| Qwen3-14b | norole | 0.99 | 0.01 | 0.99 | 0.88 | 0.05 | 0.88 | 1.00 | 0.00 | 1.00 | 0.84 | 0.18 | 0.91 | 0.85 | 0.11 | 0.88 |
|  | zeroshot | 0.99 | 0.01 | 0.99 | 0.88 | 0.09 | 0.90 | 1.00 | 0.00 | 1.00 | 0.78 | 0.18 | 0.78 | 0.82 | 0.12 | 0.81 |
|  | fewshot | 0.99 | 0.01 | 0.99 | 0.93 | 0.09 | 0.96 | 1.00 | 0.00 | 1.00 | 0.83 | 0.16 | 0.87 | 0.87 | 0.12 | 0.90 |
| Deepseek-r1-1.5b | norole | 0.93 | 0.03 | 0.93 | 0.17 | 0.23 | 0.11 | 0.97 | 0.01 | 0.98 | 0.10 | 0.10 | 0.09 | 0.11 | 0.13 | 0.08 |
|  | zeroshot | 0.90 | 0.05 | 0.90 | 0.26 | 0.25 | 0.21 | 0.91 | 0.05 | 0.91 | 0.61 | 0.25 | 0.63 | 0.32 | 0.23 | 0.30 |
|  | fewshot | 0.91 | 0.08 | 0.92 | 0.31 | 0.32 | 0.24 | 0.92 | 0.08 | 0.94 | 0.55 | 0.22 | 0.52 | 0.36 | 0.28 | 0.31 |
| Deepseek-r1-7b | norole | 0.96 | 0.02 | 0.96 | 0.55 | 0.30 | 0.61 | 0.98 | 0.02 | 0.98 | 0.38 | 0.36 | 0.41 | 0.40 | 0.33 | 0.40 |
|  | zeroshot | 0.98 | 0.02 | 0.99 | 0.74 | 0.19 | 0.82 | 0.99 | 0.02 | 0.99 | 0.76 | 0.20 | 0.82 | 0.74 | 0.17 | 0.75 |
|  | fewshot | 0.97 | 0.05 | 0.99 | 0.63 | 0.25 | 0.66 | 0.97 | 0.04 | 0.99 | 0.83 | 0.18 | 0.87 | 0.70 | 0.21 | 0.67 |
| Deepseek-r1-14b | norole | 0.99 | 0.01 | 0.99 | 0.74 | 0.38 | 0.90 | 1.00 | 0.00 | 1.00 | 0.58 | 0.38 | 0.71 | 0.63 | 0.38 | 0.79 |
|  | zeroshot | 0.98 | 0.01 | 0.98 | 0.61 | 0.23 | 0.64 | 0.98 | 0.00 | 0.98 | 0.89 | 0.15 | 0.96 | 0.70 | 0.19 | 0.69 |
|  | fewshot | 0.98 | 0.01 | 0.99 | 0.64 | 0.21 | 0.63 | 0.98 | 0.01 | 0.99 | 0.90 | 0.16 | 0.99 | 0.73 | 0.18 | 0.74 |
| Gemma3-1b | norole | 0.94 | 0.06 | 0.95 | 0.43 | 0.24 | 0.38 | 0.95 | 0.05 | 0.97 | 0.68 | 0.22 | 0.74 | 0.51 | 0.22 | 0.51 |
|  | zeroshot | 0.71 | 0.32 | 0.86 | 0.17 | 0.22 | 0.10 | 0.71 | 0.35 | 0.89 | 0.51 | 0.39 | 0.45 | 0.19 | 0.19 | 0.13 |
|  | fewshot | 0.82 | 0.22 | 0.91 | 0.26 | 0.29 | 0.10 | 0.84 | 0.23 | 0.92 | 0.52 | 0.29 | 0.43 | 0.27 | 0.26 | 0.15 |
| Gemma3-4b | norole | 0.94 | 0.06 | 0.95 | 0.43 | 0.24 | 0.38 | 0.95 | 0.05 | 0.97 | 0.68 | 0.22 | 0.74 | 0.51 | 0.22 | 0.51 |
|  | zeroshot | 0.93 | 0.07 | 0.97 | 0.44 | 0.31 | 0.39 | 0.93 | 0.08 | 0.97 | 0.91 | 0.10 | 0.95 | 0.55 | 0.30 | 0.54 |
|  | fewshot | 0.96 | 0.04 | 0.98 | 0.52 | 0.35 | 0.46 | 0.97 | 0.04 | 0.99 | 0.76 | 0.17 | 0.82 | 0.58 | 0.29 | 0.59 |
| Gemma3-12b | norole | 0.98 | 0.02 | 0.99 | 0.69 | 0.25 | 0.75 | 0.98 | 0.02 | 0.99 | 0.94 | 0.08 | 0.97 | 0.77 | 0.20 | 0.79 |
|  | zeroshot | 0.97 | 0.03 | 0.98 | 0.51 | 0.25 | 0.45 | 0.97 | 0.02 | 0.98 | 0.94 | 0.08 | 0.96 | 0.63 | 0.23 | 0.60 |
|  | fewshot | 0.98 | 0.03 | 0.99 | 0.69 | 0.22 | 0.66 | 0.98 | 0.02 | 0.99 | 0.90 | 0.11 | 0.95 | 0.77 | 0.17 | 0.79 |
| Llama2-Chinese-7b | norole | 0.95 | 0.04 | 0.96 | 0.00 | 0.00 | 0.00 | 1.00 | 0.00 | 1.00 | 0.00 | 0.00 | 0.00 | 0.00 | 0.00 | 0.00 |
|  | zeroshot | 0.92 | 0.05 | 0.92 | 0.05 | 0.05 | 0.04 | 0.96 | 0.02 | 0.97 | 0.03 | 0.03 | 0.02 | 0.03 | 0.04 | 0.03 |
|  | fewshot | 0.87 | 0.05 | 0.88 | 0.09 | 0.11 | 0.05 | 0.91 | 0.03 | 0.91 | 0.22 | 0.16 | 0.17 | 0.11 | 0.10 | 0.07 |
| Llama2-Chinese-13b | norole | 0.95 | 0.04 | 0.96 | 0.00 | 0.00 | 0.00 | 1.00 | 0.00 | 1.00 | 0.00 | 0.00 | 0.00 | 0.00 | 0.00 | 0.00 |
|  | zeroshot | 0.78 | 0.22 | 0.89 | 0.22 | 0.22 | 0.18 | 0.79 | 0.24 | 0.92 | 0.51 | 0.15 | 0.53 | 0.24 | 0.19 | 0.25 |
|  | fewshot | 0.78 | 0.10 | 0.79 | 0.19 | 0.22 | 0.08 | 0.79 | 0.10 | 0.79 | 0.78 | 0.19 | 0.84 | 0.27 | 0.27 | 0.14 |
| Llama3.1-8b | norole | 0.87 | 0.10 | 0.90 | 0.24 | 0.18 | 0.20 | 0.86 | 0.10 | 0.90 | 0.89 | 0.12 | 0.93 | 0.35 | 0.22 | 0.31 |
|  | zeroshot | 0.87 | 0.10 | 0.92 | 0.28 | 0.24 | 0.21 | 0.87 | 0.11 | 0.91 | 0.96 | 0.05 | 0.97 | 0.40 | 0.28 | 0.35 |
|  | fewshot | 0.86 | 0.11 | 0.91 | 0.25 | 0.18 | 0.19 | 0.86 | 0.12 | 0.91 | 0.94 | 0.08 | 0.97 | 0.37 | 0.21 | 0.31 |

**Table 2. Full descriptive statistics by parameter size.**

| **Model size** | **Accuracy** | | | **Precision** | | | **Specificity** | | | **Recall** | | | **F1-score** | | | **Time (Seconds)** | | |
| --- | --- | --- | --- | --- | --- | --- | --- | --- | --- | --- | --- | --- | --- | --- | --- | --- | --- | --- |
|  | **mean** | **std** | **median** | **mean** | **std** | **median** | **mean** | **std** | **median** | **mean** | **std** | **median** | **mean** | **std** | **median** | **mean** | **std** | **median** |
| 1B | 0.82 | 0.23 | 0.92 | 0.29 | 0.27 | 0.16 | 0.83 | 0.25 | 0.92 | 0.57 | 0.30 | 0.59 | 0.33 | 0.25 | 0.23 | 0.51 | 0.22 | 0.63 |
| 1.5B | 0.91 | 0.06 | 0.93 | 0.25 | 0.26 | 0.19 | 0.94 | 0.06 | 0.96 | 0.42 | 0.30 | 0.39 | 0.26 | 0.24 | 0.21 | 2.83 | 0.07 | 2.84 |
| 1.7B | 0.98 | 0.02 | 0.99 | 0.77 | 0.25 | 0.79 | 0.99 | 0.01 | 0.99 | 0.79 | 0.20 | 0.84 | 0.77 | 0.23 | 0.81 | 1.51 | 0.09 | 1.52 |
| 4B | 0.94 | 0.06 | 0.97 | 0.46 | 0.29 | 0.40 | 0.95 | 0.06 | 0.97 | 0.78 | 0.19 | 0.82 | 0.55 | 0.26 | 0.54 | 0.61 | 0.04 | 0.61 |
| 7B | 0.94 | 0.05 | 0.96 | 0.34 | 0.35 | 0.22 | 0.97 | 0.04 | 0.98 | 0.37 | 0.38 | 0.17 | 0.33 | 0.35 | 0.10 | 5.28 | 4.09 | 3.26 |
| 8B | 0.93 | 0.09 | 0.96 | 0.58 | 0.36 | 0.71 | 0.93 | 0.1 | 0.96 | 0.86 | 0.16 | 0.92 | 0.60 | 0.29 | 0.67 | 6.83 | 2.93 | 6.63 |
| 12B | 0.98 | 0.02 | 0.99 | 0.63 | 0.24 | 0.66 | 0.98 | 0.02 | 0.99 | 0.93 | 0.09 | 0.96 | 0.72 | 0.20 | 0.76 | 0.84 | 0.38 | 1.07 |
| 13B | 0.84 | 0.16 | 0.89 | 0.14 | 0.20 | 0.04 | 0.86 | 0.17 | 0.93 | 0.43 | 0.36 | 0.50 | 0.17 | 0.22 | 0.08 | 4.59 | 2.28 | 4.88 |
| 14B | 0.99 | 0.01 | 0.99 | 0.78 | 0.23 | 0.87 | 0.99 | 0.01 | 1.00 | 0.80 | 0.23 | 0.87 | 0.77 | 0.21 | 0.81 | 11.56 | 4.20 | 10.68 |

**Table 3 Comprehensive ranking of model-prompt combinations (normalized F1‑score, time, and composite score).**

| **Model** | **Prompt** | **Macro F1-score** | **Time (seconds)** | **F1 norm** | **T norm** | **score** |
| --- | --- | --- | --- | --- | --- | --- |
| Qwen3-8b | norole | 0.89 | 3.64 | 1.00 | 0.82 | 0.96 |
| Qwen3-1.7b | zeroshot | 0.85 | 1.61 | 0.96 | 0.93 | 0.95 |
| Qwen3-1.7b | fewshot | 0.83 | 1.52 | 0.93 | 0.93 | 0.93 |
| Gemma3-12b | norole | 0.77 | 0.32 | 0.87 | 0.99 | 0.9 |
| Gemma3-12b | fewshot | 0.77 | 1.13 | 0.87 | 0.95 | 0.89 |
| Qwen3-14b | fewshot | 0.87 | 11.23 | 0.98 | 0.44 | 0.85 |
| Qwen3-8b | fewshot | 0.82 | 8.04 | 0.92 | 0.60 | 0.84 |
| Deepseek-r1-7b | zeroshot | 0.74 | 3.17 | 0.84 | 0.85 | 0.84 |
| Qwen3-8b | zeroshot | 0.78 | 6.06 | 0.88 | 0.70 | 0.83 |
| Qwen3-14b | zeroshot | 0.82 | 10.13 | 0.93 | 0.49 | 0.82 |
| Qwen3-14b | norole | 0.85 | 12.80 | 0.96 | 0.36 | 0.81 |
| Deepseek-r1-7b | fewshot | 0.70 | 2.55 | 0.79 | 0.88 | 0.81 |
| Gemma3-12b | zeroshot | 0.63 | 1.07 | 0.72 | 0.96 | 0.78 |
| Qwen3-1.7b | norole | 0.63 | 1.40 | 0.71 | 0.94 | 0.77 |
| Deepseek-r1-14b | zeroshot | 0.70 | 7.05 | 0.79 | 0.65 | 0.75 |
| Gemma3-4b | fewshot | 0.58 | 0.55 | 0.66 | 0.98 | 0.74 |
| Gemma3-4b | zeroshot | 0.55 | 0.61 | 0.62 | 0.98 | 0.71 |
| Gemma3-1b | norole | 0.51 | 0.20 | 0.58 | 1.00 | 0.69 |
| Deepseek-r1-14b | norole | 0.63 | 8.32 | 0.72 | 0.59 | 0.68 |
| Gemma3-4b | norole | 0.51 | 0.66 | 0.58 | 0.98 | 0.68 |
| Deepseek-r1-14b | fewshot | 0.73 | 19.83 | 0.82 | 0.00 | 0.62 |
| Deepseek-r1-7b | norole | 0.40 | 3.34 | 0.45 | 0.84 | 0.55 |
| Deepseek-r1-1.5b | fewshot | 0.36 | 2.75 | 0.41 | 0.87 | 0.52 |
| Llama3.1-8b | norole | 0.35 | 4.29 | 0.40 | 0.79 | 0.50 |
| Deepseek-r1-1.5b | zeroshot | 0.32 | 2.91 | 0.36 | 0.86 | 0.48 |
| Gemma3-1b | fewshot | 0.27 | 0.63 | 0.31 | 0.98 | 0.48 |
| Llama3.1-8b | zeroshot | 0.37 | 7.20 | 0.42 | 0.64 | 0.47 |
| Llama2-chinese-13b | fewshot | 0.27 | 1.74 | 0.30 | 0.92 | 0.46 |
| Llama3.1-8b | fewshot | 0.40 | 11.74 | 0.45 | 0.41 | 0.44 |
| Gemma3-1b | zeroshot | 0.19 | 0.69 | 0.21 | 0.98 | 0.40 |
| Llama2-chinese-13b | zeroshot | 0.24 | 4.88 | 0.27 | 0.76 | 0.39 |
| Deepseek-r1-1.5b | norole | 0.11 | 2.84 | 0.13 | 0.87 | 0.31 |
| Llama2-chinese-7b | fewshot | 0.11 | 6.04 | 0.12 | 0.70 | 0.27 |
| Llama2-chinese-7b | zeroshot | 0.03 | 2.68 | 0.04 | 0.87 | 0.25 |
| Llama2-chinese-13b | norole | 0.00 | 7.14 | 0.00 | 0.65 | 0.16 |
| Llama2-chinese-7b | norole | 0.00 | 13.91 | 0.00 | 0.30 | 0.08 |

**Table 4. Non‑parametric tests comparing prompting strategies and parameter size groups.**

| **Comparison** | **Metric** | **Statistic** | ***P* value** |
| --- | --- | --- | --- |
| Prompt strategies | Accuracy | $\chi^{2}$ = 170.08 | **<.001** |
|  | Precision | $\chi^{2}$ = 180.74 | **<.001** |
|  | Recall | $\chi^{2}$ = 150.70 | **<.001** |
|  | Specificity | $\chi^{2}$ = 177.11 | **<.001** |
|  | F1‑score | $\chi^{2}$ = 181.57 | **<.001** |
|  | Balanced accuracy | $\chi^{2}$ = 161.82 | **<.001** |
|  | Macro‑F1 | $\chi^{2}$ = 35.00 | 0.47 |
| Parameter size groups | Time (seconds) | H = 180.82 | **<.001** |

**Table 5 Fixed effects from linear mixed models for all performance metrics.**

| **Metric** | **Effect** | ***β*** | **Std** | ***P* value** | **CI lower** | **CI upper** |
| --- | --- | --- | --- | --- | --- | --- |
| F1-score | Intercept | 0.44 | 0.09 | / | 0.26 | 0.62 |
| F1-score | Prompt-norole | -0.07 | 0.10 | .46 | -0.26 | 0.12 |
| F1-score | Prompt-zeroshot | -0.03 | 0.10 | .78 | -0.21 | 0.16 |
| F1-score | Size | 0.02 | 0.01 | **.04** | 0.00 | 0.03 |
| F1-score | Prompt-norole: Size | 0.00 | 0.01 | .84 | -0.02 | 0.02 |
| F1-score | Prompt-zeroshot: Size | 0.00 | 0.01 | .87 | -0.02 | 0.02 |
| F1-score | Group Var | 0.25 | 0.18 | .16 | -0.10 | 0.59 |
| Balanced_accuracy | Intercept | 0.76 | 0.04 | / | 0.68 | 0.84 |
| Balanced_accuracy | Prompt-norole | -0.03 | 0.05 | .54 | -0.13 | 0.07 |
| Balanced_accuracy | Prompt-zeroshot | 0.00 | 0.05 | .94 | -0.11 | 0.09 |
| Balanced_accuracy | Size | 0.01 | 0.00 | **.01** | 0.00 | 0.02 |
| Balanced_accuracy | Prompt-norole: Size | -0.01 | 0.01 | .36 | -0.02 | 0.01 |
| Balanced_accuracy | Prompt-zeroshot: Size | 0.00 | 0.01 | .65 | -0.01 | 0.01 |
| Balanced_accuracy | Group Var | 0.08 | 0.07 | .25 | -0.05 | 0.20 |
| Accuracy | Intercept | 0.91 | 0.03 | / | 0.86 | 0.96 |
| Accuracy | Prompt-norole | 0.02 | 0.03 | .44 | -0.04 | 0.09 |
| Accuracy | Prompt-zeroshot | -0.03 | 0.03 | .31 | -0.10 | 0.03 |
| Accuracy | Size | 0.00 | 0.00 | .39 | 0.00 | 0.01 |
| Accuracy | Prompt-norole: Size | 0.00 | 0.00 | .89 | -0.01 | 0.01 |
| Accuracy | Prompt-zeroshot: Size | 0.00 | 0.00 | .41 | 0.00 | 0.01 |
| Accuracy | Group Var | 0.10 | 0.08 | .22 | -0.06 | 0.26 |
| Precision | Intercept | 0.41 | 0.10 | / | 0.22 | 0.60 |
| Precision | Prompt-norole | -0.08 | 0.10 | .45 | -0.28 | 0.12 |
| Precision | Prompt-zeroshot | -0.02 | 0.10 | .85 | -0.22 | 0.18 |
| Precision | Size | 0.01 | 0.01 | .08 | 0.00 | 0.03 |
| Precision | Prompt-norole: Size | 0.00 | 0.01 | .79 | -0.02 | 0.03 |
| Precision | Prompt-zeroshot: Size | 0.00 | 0.01 | .92 | -0.02 | 0.02 |
| Precision | Group Var | 0.23 | 0.19 | .23 | -0.15 | 0.61 |
| Recall | Intercept | 0.59 | 0.08 | **/** | 0.44 | 0.74 |
| Recall | Prompt-norole | -0.09 | 0.10 | .36 | -0.29 | 0.10 |
| Recall | Prompt-zeroshot | 0.03 | 0.10 | .77 | -0.17 | 0.22 |
| Recall | Size | 0.02 | 0.01 | **.02** | 0.00 | 0.03 |
| Recall | Prompt-norole: Size | -0.01 | 0.01 | .31 | -0.03 | 0.01 |
| Recall | Prompt-zeroshot: Size | -0.01 | 0.01 | .43 | -0.03 | 0.01 |
| Recall | Group Var | 0.05 | 0.05 | .32 | -0.05 | 0.15 |
| Specificity | Intercept | 0.92 | 0.03 | **/** | 0.87 | 0.97 |
| Specificity | Prompt-norole | 0.03 | 0.03 | .39 | -0.04 | 0.10 |
| Specificity | Prompt-zeroshot | -0.04 | 0.03 | .27 | -0.10 | 0.03 |
| Specificity | Size | 0.00 | 0.00 | .58 | 0.00 | 0.01 |
| Specificity | Prompt-T.norole: Size | 0.00 | 0.00 | .78 | -0.01 | 0.01 |
| Specificity | Prompt-zeroshot: Size | 0.00 | 0.00 | .34 | 0.00 | 0.01 |
| Specificity | Group Var | 0.06 | 0.06 | .28 | -0.05 | 0.18 |
