# Supplementary material for: The Performance of Large Language Models in Extracting Intestinal Symptoms From Electronic Health Records: Retrospective Observational Study
Source: J Med Internet Res. 2026 Aug 26;28:e98580. doi: 10.2196/98580 (PMC13509444; doi:10.2196/98580)
Supplement: Supplementary file 6 [file jmir-v28-e98580-s005.docx]

**Figure 1 Confusion matrices for model Qwen3-1.7b across six target symptoms.**

Each 2×2 matrix displays the number of predictions for the four possible outcomes: true positive (TP), false negative (FN), false positive (FP), and true negative (TN). The x-axis of each subplot indicates the predicted label (Positive/Negative), and the y-axis indicates the true label (True/False). All values are averaged over the available test samples for each model-symptom combination; the underlying dataset consists of manually annotated notes from healthcare information platform, as detailed in Methods.

**Figure 2 Confusion matrices for model Qwen3-8b across six target symptoms.**

Each 2×2 matrix displays the number of predictions for the four possible outcomes: true positive (TP), false negative (FN), false positive (FP), and true negative (TN). The x-axis of each subplot indicates the predicted label (Positive/Negative), and the y-axis indicates the true label (True/False). All values are averaged over the available test samples for each model-symptom combination; the underlying dataset consists of manually annotated notes from healthcare information platform, as detailed in Methods.

**Figure 3 Confusion matrices for model Qwen3-14b across six target symptoms.**

Each 2×2 matrix displays the number of predictions for the four possible outcomes: true positive (TP), false negative (FN), false positive (FP), and true negative (TN). The x-axis of each subplot indicates the predicted label (Positive/Negative), and the y-axis indicates the true label (True/False). All values are averaged over the available test samples for each model-symptom combination; the underlying dataset consists of manually annotated notes from healthcare information platform, as detailed in Methods.

**Figure 4 Confusion matrices for model Deepseek-r1-1.5b across six target symptoms.**

Each 2×2 matrix displays the number of predictions for the four possible outcomes: true positive (TP), false negative (FN), false positive (FP), and true negative (TN). The x-axis of each subplot indicates the predicted label (Positive/Negative), and the y-axis indicates the true label (True/False). All values are averaged over the available test samples for each model-symptom combination; the underlying dataset consists of manually annotated notes from healthcare information platform, as detailed in Methods.

**Figure 5 Confusion matrices for model Deepseek-r1-7b across six target symptoms.**

Each 2×2 matrix displays the number of predictions for the four possible outcomes: true positive (TP), false negative (FN), false positive (FP), and true negative (TN). The x-axis of each subplot indicates the predicted label (Positive/Negative), and the y-axis indicates the true label (True/False). All values are averaged over the available test samples for each model-symptom combination; the underlying dataset consists of manually annotated notes from healthcare information platform, as detailed in Methods.

**Figure 6 Confusion matrices for model Deepseek-r1-14b across six target symptoms.**

Each 2×2 matrix displays the number of predictions for the four possible outcomes: true positive (TP), false negative (FN), false positive (FP), and true negative (TN). The x-axis of each subplot indicates the predicted label (Positive/Negative), and the y-axis indicates the true label (True/False). All values are averaged over the available test samples for each model-symptom combination; the underlying dataset consists of manually annotated notes from healthcare information platform, as detailed in Methods.

**Figure 7 Confusion matrices for model Gemma3-1b across six target symptoms.**

Each 2×2 matrix displays the number of predictions for the four possible outcomes: true positive (TP), false negative (FN), false positive (FP), and true negative (TN). The x-axis of each subplot indicates the predicted label (Positive/Negative), and the y-axis indicates the true label (True/False). All values are averaged over the available test samples for each model-symptom combination; the underlying dataset consists of manually annotated notes from healthcare information platform, as detailed in Methods.

**Figure 8 Confusion matrices for model Gemma3-4b across six target symptoms.**

Each 2×2 matrix displays the number of predictions for the four possible outcomes: true positive (TP), false negative (FN), false positive (FP), and true negative (TN). The x-axis of each subplot indicates the predicted label (Positive/Negative), and the y-axis indicates the true label (True/False). All values are averaged over the available test samples for each model-symptom combination; the underlying dataset consists of manually annotated notes from healthcare information platform, as detailed in Methods.

**Figure 9 Confusion matrices for model Gemma3-12b across six target symptoms.**

Each 2×2 matrix displays the number of predictions for the four possible outcomes: true positive (TP), false negative (FN), false positive (FP), and true negative (TN). The x-axis of each subplot indicates the predicted label (Positive/Negative), and the y-axis indicates the true label (True/False). All values are averaged over the available test samples for each model-symptom combination; the underlying dataset consists of manually annotated notes from healthcare information platform, as detailed in Methods.

**Figure 10 Confusion matrices for model Llama2-Chinese-7b across six target symptoms.**

Each 2×2 matrix displays the number of predictions for the four possible outcomes: true positive (TP), false negative (FN), false positive (FP), and true negative (TN). The x-axis of each subplot indicates the predicted label (Positive/Negative), and the y-axis indicates the true label (True/False). All values are averaged over the available test samples for each model-symptom combination; the underlying dataset consists of manually annotated notes from healthcare information platform, as detailed in Methods.

**Figure 11 Confusion matrices for model Llama2-Chinese-13b across six target symptoms.**

Each 2×2 matrix displays the number of predictions for the four possible outcomes: true positive (TP), false negative (FN), false positive (FP), and true negative (TN). The x-axis of each subplot indicates the predicted label (Positive/Negative), and the y-axis indicates the true label (True/False). All values are averaged over the available test samples for each model-symptom combination; the underlying dataset consists of manually annotated notes from healthcare information platform, as detailed in Methods.

**Figure 12 Confusion matrices for model Llama3.1-8b across six target symptoms.**

Each 2×2 matrix displays the number of predictions for the four possible outcomes: true positive (TP), false negative (FN), false positive (FP), and true negative (TN). The x-axis of each subplot indicates the predicted label (Positive/Negative), and the y-axis indicates the true label (True/False). All values are averaged over the available test samples for each model-symptom combination; the underlying dataset consists of manually annotated notes from healthcare information platform, as detailed in Methods.
